# Contribution of Range‐Wide and Short‐Scale Chemical Soil Variation to Local Adaptation in a Tropical Montane Forest Tree

**DOI:** 10.1111/eva.70116

**Published:** 2025-07-09

**Authors:** Sebastián Arenas, Jorge Cruz‐Nicolás, Gustavo Giles‐Pérez, Josué Barrera‐Redondo, Verónica Reyes‐Galindo, Alicia Mastretta‐Yanes, Erika Aguirre‐Planter, Luis E. Eguiarte, Juan Pablo Jaramillo‐Correa

**Affiliations:** ^1^ Departamento de Ecología Evolutiva, Instituto de Ecología Universidad Nacional Autónoma de México Ciudad de México México; ^2^ Department of Plant Protection Biology Swedish University of Agricultural Sciences Alnarp Sweden; ^3^ Instituto de Geografía Universidad Nacional Autónoma de México Ciudad de México México; ^4^ Posgrado en Ciencias Biológicas, Universidad Nacional Autónoma de México Ciudad de México México; ^5^ Department of Biotechnology and Biochemistry Center for Research and Advanced Studies (Cinvestav) Irapuato Gto Mexico; ^6^ CONACYT‐CONABIO, Comisión Nacional Para el Conocimiento y Uso de la Biodiversidad Ciudad de México México

**Keywords:** edaphic variation, genomic diversity, geographic scales, Mexican fir

## Abstract

Local adaptation is a fundamental process that allows populations to thrive in their native environment, often increasing genetic differentiation with neighboring stands. However, detecting the molecular basis and selective factors responsible for local adaptation remains a challenge, particularly in sessile, non‐model species with long life cycles, such as forest trees. Local adaptation in trees is not only modeled by climatic factors, but also by soil variation. Such variation depends on dynamic geological and ecological processes that generate a highly heterogeneous selective mosaic that may differentially condition tree adaptation both at the range‐wide and local scales. This could be particularly manifest in species inhabiting mountain ranges that were formed by diverse geological events, like sacred fir (*Abies religiosa*), a conifer endemic to the mountains of central Mexico. Here, we used landscape genomics approaches to investigate how chemical edaphic variation influences the genetic structure of this species at the range‐wide and local scales. After controlling for neutral genetic structure, we performed genotype‐environment associations and identified 49 and 23 candidate SNPs at the range‐wide and local scales, respectively, with little overlap between scales. We then developed polygenic models with such candidates, which accounted for ~20% of the range‐wide variation in soil *Ca* ^2+^ concentration, electric conductivity (*EC*), and *pH*, and for the local variation in soil *EC* and organic carbon content (*OC*). Spatial Principal Component Analyses further highlighted the role of geography and population isolation in explaining this genetic‐soil co‐variation. Our findings reveal that local adaptation in trees is the result of an intricate interaction between soil chemical properties and the local population's genetic makeup, and that the selective factors driving such adaptation greatly vary and are not necessarily predictable across spatial scales. These results highlight the need to consider edaphic variation in forest genetic studies (including common garden experiments) and in conservation, management and assisted migration programs.

## 1 Introduction

Understanding how the environment influences the distribution of genetic variation is a major goal in evolutionary ecology and biogeography (Sanmartín and Ronquist 2004; Rellstab et al. 2017; Martins et al. 2018). Environmental selective pressures often vary in space and time, causing populations to evolve traits that are advantageous under local conditions (Blanquart et al. 2013; Lascoux et al. 2016; Guerrero et al. 2018). The study of local adaptation is paramount for predicting species' evolutionary responses to present and future environmental changes (Nielsen et al. 2011; Prunier et al. 2013), from which we can guide conservation and management programs (Savolainen et al. 2013; Oddou‐Muratorio et al. 2020; Neophytou et al. 2022).

Identifying adaptive processes in natural landscapes can be challenging because the main environmental agents that have historically shaped genetic variability are mostly unknown (Manel et al. 2010; Aitken et al. 2008; Flanagan et al. 2018). For instance, genetic divergence across a heterogeneous landscape is often driven by the environmental conditions at different geographic and temporal scales that either impose selective pressures on individuals or limit gene flow (Mckown et al. 2014; Dalongeville et al. 2018). Such processes can be further amplified by historical demographic factors (genetic drift) that can be correlated to environmental variation (Zellmer and Knowles 2009), such as the one observed during the Pleistocene glacial cycles (Excoffier et al. 2009; Sork et al. 2013).

During the last two decades, disentangling the evolutionary factors that shape genetic diversity has been facilitated by next‐generation sequencing and novel, powerful statistical approaches, particularly for understanding the genetic basis of local adaptation at various landscape scales (Manel et al. 2010; Forester et al. 2016). This is especially important for long‐lived species with large genomes, like forest trees, which are at the base of vast terrestrial ecosystems and possess important economic value (Brockerhoff et al. 2008). Understanding the patterns behind local forest adaptation can thus help optimize seed supply, reforestation efforts, and other forest management applications (Aitken and Whitlock 2013; Aitken and Bemmels 2016; Martins et al. 2018). Such a knowledge can further contribute to forest persistence under current and future environmental challenges (Jump and Peñuelas 2005; Savolainen et al. 2007; Alberto et al. 2013; Sork et al. 2013; Isabel et al. 2020).

Recently, landscape genomics approaches have been implemented to identify potential candidate genes that may respond to local selective pressures (Riordan et al. 2016; Scotti et al. 2016; Talbot et al. 2017). Forest trees are particularly suitable for this kind of study, as they grow over long periods of time in heterogeneous environments. Evidence exists that trees are indeed locally adapted at both the range‐wide (e.g., Eckert et al. 2012 in *Pinus*) and local spatial scales (~20 km) (e.g., Eckert et al. 2012 in *Pinus*; Pluess et al. 2016 in *Fagus*; Brousseau et al. 2020 in *Eperua falcata*; Zimmermann et al. 2025 in *Quercus*). Landscape genetics studies in forest trees have indeed shown that local adaptation can arise independently through similar selective forces acting on isolated genetic lineages (Orsini et al. 2013; Prunier et al. 2013; Riordan et al. 2016) or within the same lineage that evolves in a heterogeneous landscape (e.g., Scotti et al. 2016; Rellstab et al. 2017). However, how gene flow and adaptive processes interact across nested spatial or temporal scales remains unclear, as does the extent to which polymorphisms identified at one scale predict adaptation at a larger or smaller scale. If genotype‐environment associations hold across scales, strong, shared selective pressures may be shaping adaptation. In contrast, shifts between scales suggest context‐dependent local adaptation. For species with high levels of gene flow, like conifers, the balance between this homogenizing force and the local selective constraints may condition our ability to predict adaptive responses, depending on the scale at which we are evaluating such changes.

When compared with climate factors, soil variation has been somehow overlooked in local adaptation studies in forest trees (Schweitzer et al. 2011; Purahong et al. 2016). However, edaphic traits are capital for tree establishment and survival, and they can be highly heterogeneous along a landscape, driving genetic divergence at multiple geographic scales (including the microenvironmental level) (Mckown et al. 2014; Gugger et al. 2021). This spatial heterogeneity may exert differential selective pressures on populations, generating local adaptations that are not necessarily observed at broader scales (Gugger et al. 2021). Thus, it is likely that the dynamic geological and ecological processes that have driven soil formation are related to the spatial distribution of genetic variation in forest trees. Indeed, some range‐wide studies have detected associations between edaphic variables, such as the nitrogen and phosphorus concentration in the soil, soil pH, and patterns of genetic divergence in trees (Plomion et al. 2016; Collevatti et al. 2019; Ellis and Jonågren 2024). Such associations have also been observed at finer spatial scales, including a differential capacity to use the various forms of nitrogen available in edaphic microsites between genotypes (Guerrero et al. 2018; Arenas et al. 2021). Assessing the consistency of these associations across spatial scales will provide valuable information on the predictability of local adaptation across the landscape and help optimizing “gain size” in landscape genomics studies in forest trees.

The Trans‐Mexican Volcanic Belt (TMVB) is a complex mountain range located in central Mexico that was formed through a mosaic of geological processes during the last 65 My (Siebert et al., 2002; Gómez‐Tuena et al. 2005). These processes have generated a large number of volcanic structures since the early Miocene, particularly during the last 2.5 Myr (late Pliocene and Pleistocene; Gómez‐Tuena et al. 2005; Ferrari et al. 2012), during which most of the massive stratovolcanoes (> 3500 m) of the region began their formation. These volcanoes are now covered by large temperate forests, whose dominant species usually exhibit a strong population genetics structure (e.g., Herrera‐Arroyo et al. 2013; Giles‐Pérez et al. 2022; Izaguirre‐Toriz et al. 2024). It is believed that such a structure has been modelled, at least in part, by the soil characteristics of the TMVB, which has likely changed innumerable times and at different spatial and temporal scales, among others because of volcanic and glacial activities (Gómez‐Tuena et al. 2005; Ferrari et al. 2012).

In the present study, we focused on sacred fir (*Abies religiosa* (Kunth) Schltdl. and Cham.), one of the dominant taxa of the montane forests along the TMVB, at elevations ranging from 2400 to 3600 m asl (Castellanos‐Acuña et al. 2014). This species plays a key ecological role in water retention and soil stabilization, and as the overwintering habitat of the monarch butterfly ( *Danaus plexippus* ; Sáenz‐Romero et al. 2024). Sacred fir has a strong genetic and morphological differentiation along both longitudinal and altitudinal gradients (e.g., Ortiz‐Bibian et al. 2017; Cruz‐Nicolás et al. 2019; Giles‐Pérez et al. 2022), which might be due, at least in part, to local adaptation. Understanding how such structure has been modeled is essential for developing conservation and management strategies (Sáenz‐Romero et al. 2012), given that climate projections indicate that its suitable habitat could decrease by up to 92% by the end of the century (Heredia‐Bobadilla 2012).

Here, we explored how the balance between gene flow and local edaphic selection varies between spatial scales. To do so, we compared the associations detected between the genetic composition of sacred fir populations and soil chemical properties at both a range‐wide and a local scale. At the range‐wide scale, we expected strong genetic differences between stands, which could be explained by a combination of population isolation and selection driven by edaphic variation. At the local scale, we expected a more subtle genetic structure produced by the homogenizing effect of pollen‐mediated gene flow (Ortiz‐Bibian et al. 2017; Paluch et al. 2019). However, we also anticipated that the high edaphic heterogeneity observed at this scale may also favor local adaptation (Méndez‐González et al. 2017; Zimmermann et al. 2025).

More specifically, we aimed to (1) identify candidate SNPs at both spatial scales using GEA (Genotype‐environment associations), (2) compare and look for candidate SNP repeatability between scales, and (3) develop polygenic models to predict the variation in one scale using the candidates detected at the other scale. This multiscale approach should help improve our ability to predict how forests will respond to future environmental changes and contribute to the discussion regarding one of the most recurrent challenges in landscape genetics studies: how to define an appropriate grain size for detecting local adaptation (Manel et al. 2010; Forester et al. 2016).

## 2 Materials and Methods

### 2.1 Plant Material, Extraction, Sequencing, Assembly, and SNP Calling

**FIGURE 1:**
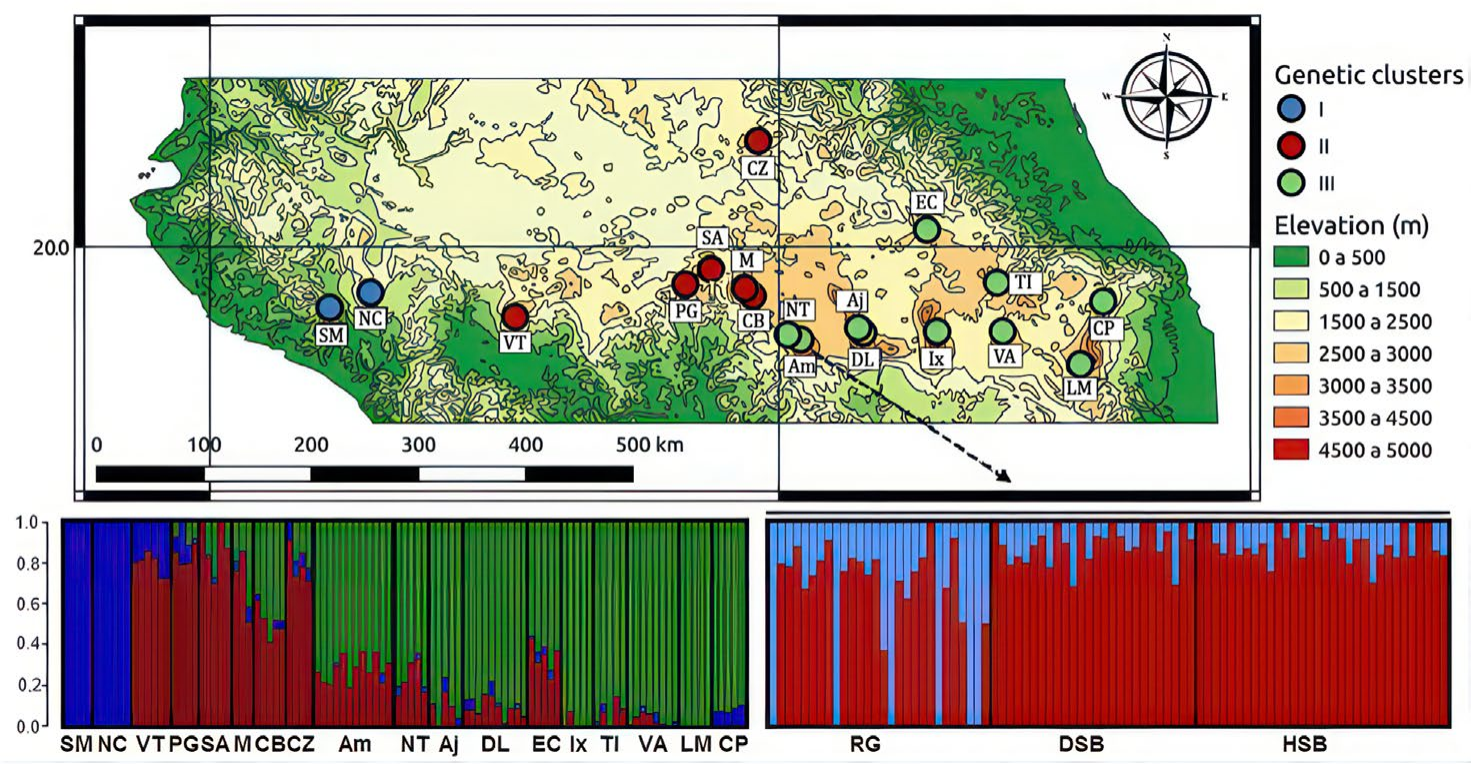
Top. Geographic location of sacred fir (*Abies religiosa*) populations surveyed at the range‐wide and local scales (location indicated with a dashed arrow). Bottom. Contribution of identified genetic clusters to each population and individual (left: *K* = 3 at the range‐wide scale; right: *K* = 2 at the local scale). The full names of the abbreviated populations are provided in Table 1 for the wide‐range scale and in Suppl. Table 1 for the local scale. Am, Amanalco; CZ, Cerro Zamorano; CB, Cerro Blanco; CP, Cofre del Perote; DL, Desierto de Leones; Aj, Ajusco; EC, El Chico; Ix, Ixtapalucan; LM, La Malinche; M, Monarca; NC, Nevado de Colima; NT, Nevado de Toluca; PG, Puerta Garnica; SA, San Andrés; SM, Sierra Manantlán; Tl, Tlaxco; VA, Volcán Atlitzín; VT, Volcán Tancítaro; RG, Rincón de Guadalupe; HSB, Hight San Bartolo; DSB, Down San Bartolo.

For studying range‐wide variation, we selected 113 individuals from previous sampling efforts across the natural distribution of *A. religiosa* (Aguirre‐Planter et al. 2000; Cruz‐Nicolás et al. 2019; Giles‐Pérez et al. 2022; that is, range‐wide scale; see Figure 1A and Table 1 for information on sampling in each population). These needle samples were collected between 1996 and 2019 for adult cone‐bearer trees separated by at least 30 m from each other at 18 fir populations that were at least 6 km apart. Samples are all preserved at −80°C in the germplasm bank at the Institute of Ecology, Universidad Nacional Autónoma de Mexico (IE‐UNAM).

**TABLE 1:** Sample size, coordinates, elevation, and genetic diversity estimates for each of the 18 sacred fir (*Abies religiosa*) populations analyzed, and for the three range‐wide genetic clusters obtained with Admixture (see Figure 1 and Table S2).

Population	*N*	*N*_ES_	Longitude	Latitude	Elevation	*π*	*H*_*O*_	*H*_E_
Sierra Manantlán (SM)	6	4	−103.63	19.63	2515	0.026	0.073	0.091
Ajusco (Aj)	6	5	−99.23	19.22	3369	0.046	0.188	0.200
Desierto de Leones (DL)	10	10	−99.30	19.29	3474	0.042	0.193	0.198
El Chico (EC)	6	5	−98.7	20.15	2940	0.043	0.188	0.193
Nevado de Colima (NC)	6	6	−103.59	19.597	3276	0.029	0.127	0.139
Nevado de Toluca (NT)	5	5	−99.81	19.18	3387	0.040	0.190	0.199
Ixtapalucan (Ix)	5	5	−98.61	19.25	3236	0.044	0.196	0.201
Cerro Blanco (CB)	5	5	−100.24	19.57	3416	0.044	0.192	0.198
Puerta Garnica (PG)	4	4	−100.82	19.67	2913	0.040	0.174	0.184
Volcán Tancítaro (VT)	6	6	−102.32	19.38	3030	0.031	0.153	0.164
Monarca (M)	6	3	−100.23	19.57	3516	0.049	0.196	0.208
San Andrés (SA)	5	5	−100.59	19.80	3237	0.042	0.180	0.208
Amanalco (Am)	12	12	−99.92	19.23	3170	0.044	0.196	0.204
Volcán Atlitzin (VA)	8	8	−97.35	18.97	3060	0.049	0.186	0.200
Cerro Zamorano (CZ)	5	4	−100.18	20.93	3156	0.040	0.187	0.191
Tlaxco (Tl)	5	5	−98.08	19.68	2760	0.045	0.192	0.198
La Malinche (LM)	6	5	−98.04	19.26	3358	0.043	0.181	0.184
Cofre del Perote (CP)	7	5	−97.15	19.52	3510	0.050	0.177	0.190
Cluster I	—	11	—	—	—	0.031	0.113	0.144
Cluster II	—	24	—	—	—	0.043	0.184	0.201
Cluster III	—	67	—	—	—	0.047	0.189	0.204
Whole sample	113	102				0.046	0.186	0.209

*Note:* Tajima's D. N corresponds to the number of originally selected individuals per population and NES to the number of individuals that were efficiently sequenced.

Abbreviations: *π*, Nucleotide diversity; *H*_*E*_, expected heterozygosity; *H*_*O*_, observed heterozygosity.

For the local scale variation, we selected samples from three populations resulting from natural regeneration located in the central area of the distribution of *A. religiosa* (*n* = 95, Figure S1 and Table S1). These stands differ in elevation (between ~2500 and ~3400 m.a.s.l), soil and vegetation composition, and were separated by a maximum of 7.4 km from each other (Arenas et al. 2021). For both this and the range‐wide scale, we made sure to include only individuals resulting from the natural regeneration of the native forest and avoided those introduced by management programs (see Arenas et al. 2021).

Total DNA was extracted with the Qiagen DNeasy Plant Mini Kit. DNA integrity was verified with a 1% agarose gel electrophoresis and quantified using QubitTM V 3.0. After being normalized at a concentration of 20–25 ng/μL, DNA was sequenced on the genomic analysis platform at the Institute of Integrative Biology and Systems at Université Laval (http://www.ibis.ulaval.ca/en/services‐2/genomic‐analysis‐platform/). Libraries were prepared following Poland et al. (2012), after digestion with restriction enzymes *MspI* (C|CGG) and *PstI* (TGCA|G). Sequencing was performed on an Illumina HiSeq2500 system, which produced ~100 bp single‐end reads. Read quality was determined with *FASTQC*, before and after demultiplexing, and quality filtering. These last steps, together with *de novo* assembly, read alignment and SNP calling were performed with the *IPYRAD* v0.7.23 pipeline (Eaton 2014). Assembly parameters included a clustering threshold of 0.9, a minimum majority rule depth of 100,000, a minimum sequencing depth of 8, and a maximum barcode mismatch of 0. We then used *PLINK* v1.07 (Purcell 2010) to remove monomorphic reads, variants with missing call rates above 20% or that were in Hardy–Weinberg disequilibrium within populations (*p*‐value < 1 × 10^−6^; Minamikawa et al. 2018), and samples with minimum allele frequencies (MAF) below 5%. Given that complete datasets are required for assessing genotype‐environment associations, we imputed missing genotypes with *TASSEL* v.5 (Bradbury et al. 2007) using LD and the *K*‐nearest neighbor (Beretta and Santaniello 2016).

### 2.2 Genetic Diversity, Population Structure, and Differentiation

The final dataset included 1585 SNPs that were successfully genotyped for 189 individuals; these comprised 102 trees for the range‐wide scale study and 87 plants for the local‐scale survey (Table 1 and Table S1). These were used to estimate the mean observed (*Ho*) and expected heterozygosities (*He*) and the nucleotide diversity (π) per population, with R package *hierfstat* (Goudet and Jerome et al., 2015), *PLINK* v1.04 (Purcell 2010), and *DNAsp* v.5 (Rozas et al. 2017). Population structure was inferred through a Principal Components Analysis (PCA), in *SNPRelate* (Zheng et al. 2012), and *ADMIXTURE* (Alexander and Lange 2015). For this last approach, we performed 10 replicate runs for *k* values ranging from 1 to 10, using cross‐validation for choosing the most likely value of *k* (Alexander et al. 2009; Fatokun et al. 2018), and *Q*‐plots for visualizing results.

### 2.3 Collection and Processing of Soil Environmental Variables

We collected high‐resolution data for nine soil chemical traits per population (Cruz‐Cárdenas et al. 2014), which have been found to account for most of the forest soil variability along the TMVB (Peña‐Ramírez et al. 2015). These data are the result of a comprehensive interpolation at a national scale (1:1,000,000) of 4400 random soil samples from evenly distributed points across Mexico's continental surface (approximately 1,949,359 km^2^; Ortiz‐Solorio and Gutierrez‐Castorena 2001). Each of these samples was taken from the top 20 cm of the soil and was evaluated for the electrical conductivity (EC), the content of both organic carbon (OC; in kg m^−2^) and organic matter (OM), and the concentration of *Ca* ^*2*+^, *K* ^+^, *Mg* ^*2*+^, and *Na* ^+^. The final datasets were generated from models (i.e., exponential, pentaspherical and spherical) selected through rigorous 10‐fold cross‐validation (Cruz‐Cárdenas et al. 2014) at a spatial resolution of approximately 1 km^2^, which is compatible with large‐scale environmental studies.

We obtained values for the location of each fir population using the extract function in the *raster* package in R (Hijmans 2024). Highly correlated soil variables (Pearson's *r* ≥ 0.75) were removed through pairwise multicollinearity analyses using the *corrplot* package (Wickham 2019). We performed the multivariate and univariate normality tests using Royson's test and Anderson‐Darling test in the *MVN* package (Korkmaz et al. 2014). The integration of these geostatistically derived datasets allowed us to maintain the scientific integrity and reproducibility of our work while eliminating the need for additional field sampling or laboratory analyses.

### 2.4 Gene–Environment Association (GEA) Analyses

We independently searched for genotype–chemical soil associations at both geographic scales (range‐wide and local) using three conceptually different genetic‐environmental association (GEA) methods (*LFMM*, *SAMβADA* and *BAYESCENV*). *LFMM* is a Bayesian regression MCMC algorithm that models random effects, using population history and isolation‐by‐distance, as unobserved (latent) factors (Frichot et al. 2013). This approach has been shown to be effective when selection is weak and populations have a complex hierarchical structure (Lotterhos and Whitlock 2015; Rellstab et al. 2015). Based on population structure analyses (see Results), we assumed two (at the local scale) and three (at the range‐wide scale) latent factors (see Results) and performed five runs of 50,000 iterations for each chemical soil variable after an initial burn‐in 5000 steps. We calculated median *z*‐scores and adjusted *p*‐value (*Q*) using a genomic inflation factor (λ) procedure as in (Devlin and Roeder 1999), and retained candidate SNPs after applying an FDR (false discovery rate) of 0.05 with the Benjamini‐Hochberg procedure (François et al. 2016).

*SAMβADA* is based on a logistic regression model that incorporates both spatial autocorrelation and neutral genetic structure, considering the geographic coordinates of populations and a pre‐defined number of genetic clusters (Frichot et al. 2013). We assumed the same number of clusters as for *LFMM* and performed analyses for all possible gene–environment pairs. After performing *G‐score tests* (Duruz et al. 2019), we retained loci with significant *Q*‐values after Bonferroni correction (equivalent to *p*‐value = 3.16 × 10^−5^).

*BAYESCENV* detects putative signals of local adaptation by combining an *F*_*ST*_ outlier approach with associations to the environmental variation of populations (De Villemereuil et al. 2013). We calculated environmental distances for each soil variable as the population value of each variable subtracted by the average of all populations. We ran two independent MCMC analyses with 20 initial pilot runs of 10,000 generations for parameter fine‐tuning, followed by a main run; this had an initial burn‐in of 100,000 generations, after which samples were taken every 20 generations for 100,000 generations. We confirmed the convergence between runs by using the Gelman and Rubin statistic (Gelman and Rubin 1992) and retained loci with *Q*‐values below 0.05 (Storey and Tibshirani 2003). The flanking ~80 bp on each side of the candidate SNPs detected by all three methods were blasted for nucleotide similarity against the *TodoFirGene* transcriptome database (https://forestgen.ffpri.go.jp/en/info_todomatsu.html) (Ueno et al. 2018).

As a first approximation to identify putative differential effects of stochastic and adaptive forces on population structure, we performed Discriminant Analyses of Principal Components (DAPCs) with the identified candidates and with non‐retained loci at both scales; we used *adegenet* v.2.1.0 (Jombart 2008) and assumed cluster values (*K*) of 1–20. We determined the most likely *K*‐value for each dataset and scale by running 60 iterations of the ‘*find.clusters*’ function (Jombart 2008) and averaged Bayesian information criterion (BIC) values across iterations (Miller et al. 2020). We also simulated 100 pairwise *F*_ST_ matrices among genetic lineage pairs using both the candidate and non‐candidate datasets in *ARLEQUIN* 3.5.2 (Excoffier and Lischer 2010) with 10,000 permutations and retained their values and *p*‐value.

### 2.5 Partitioning Total SNP Variation

To assess the relative contribution of demographic history and chemical soil traits on the distribution of genetic variation, we estimated the amount of genetic diversity attributable to chemical soil differences using polygenic models built through distance‐based redundancy analyses (dbRDA). dbRDA is a form of ordination that allows assessing the explanatory power of multivariate predictors (in this case, chemical soil traits) that best explain linear combinations of the response variables (genotypes; Legendre and Legendre 2012). As with other forms of eigen‐analyses, predictors are summarized in canonical axes that are orthogonal to each other (Gibson and Moyle 2020). Here, these axes represented the genotypic variance of the candidate SNPs, which are also correlated (explained) to selected chemical soil predictors (i.e., those with a variance inflation factor (VIF) lower than 3). Additionally, we incorporated the principal components (PCA) of the SNPs as conditioning variables to account for population structure (see above). To better capture the edapho‐chemical distance between samples, we used the non‐Euclidean Bray‐Curtis distance. We performed dbRDAs at both scales using the *dbrda* function in vegan, v. 2.5.2 (Oksanen et al. 2019) by applying a forward selection of predictors, which allowed optimizing the adjusted coefficient of determination (*R* ^*2*^) of the model (Peres‐Neto et al. 2006). We tested the significance of each final canonical/constrained axis with the *anova.cca* function, by running 999 permutations of the genotype matrix as in Forester et al. (2018). For each scale, we performed this analysis using the candidates retained in the genotype‐environment associations and a set of non‐retained loci that matched the number and MAF of the candidates (Segovia et al. 2020). For accounting for possible biases, we constructed 20 random subsets of such non‐candidate loci with the *sample()* function of R (i.e., there were 40 subsets in total). For each analysis at each scale, we first selected the most important explanatory variables, after eliminating co‐linear traits and those with a variance inflation factor (VIF) lower than 3 (Capblancq et al. 2018; Forester et al. 2018), and calculated the total genotypic variance explained by these predictors. Then, to test replicability between spatial scales, we developed predictive models for the range‐wide dataset using the candidate SNP retained at the local scale and *vice versa*. We assumed that if the associations were not spurious or produced by stochastic population processes, the proportion of variation calculated with candidate loci would always be larger than that obtained with random data subsets.

### 2.6 Spatial Interpolation of Candidate SNP Variation

To analyze the geographic distribution of the candidate SNP variation, we performed a Spatial Principal Component Analysis (sPCA) on all candidates detected with all methods at both scales (i.e., 49 loci at the range‐wide level and 23 at the local scale) using *adegenet* v.2.1.0 (Jombart 2008). sPCA is a spatially explicit multivariate method that produces scores (or *lagged scores*) that summarize the genetic variability and spatial structure among individuals (Jombart 2008). These scores are presented as canonical axes, which are linear combinations of genetic variables and represent multilocus geographic clines with polygenic effects (Segovia et al. 2020). To determine the role of soil variation in driving such clines, we interpolated the *lagged scores* of the first two environmental axes and each soil predictor on a 10× m resolution grid that covered the whole species range using *MATLAB* (The MathWorks Inc., 2022). We repeated this analysis by using the minor allele frequencies (MAF) of the candidates that had the highest effects on the sPCA to assess if they were more associated with the soil chemical variation than those contributing the least to the sPCA. Finally, we carried out multiple stepwise linear regressions using the *lm*() function in R, using individuals as replicates, multilocus clines as response variables, and chemical soil predictors (including the retained environmental axes) as explanatory variables to pinpoint individual variables that best explained the spatial distribution of candidate loci. Independent models were built for each geographic scale.

## 3 Results

### 3.1 Data Filtering, Genetic Diversity, and Population Genetic Structure

We obtained an average of 2,803,267 raw reads per individual, with an average length of 82 bp per read. After quality filtering, 31,462 consensus reads were retained and assembled *de novo* for identifying 373,267 SNPs. After posterior quality filtering (including Hardy–Weinberg disequilibrium, minimal allele frequencies below 5% and consensus read present in less than 80% of individuals), we retained 1587 of these high‐quality SNPs that were genotyped for 102 individuals sampled for the range wide‐scale study and 87 trees for the local scale study.

Following cross‐validation (Table S2), admixture results revealed three genetic clusters (*k*‐value) at the range‐wide scale and two groups at the local scale (Figure 1 and Table S2A). At the range‐wide scale, the westernmost populations (SM and NC; Cluster I) formed a clearly distinct group, while stands at the center (CB, CZ, PG, VT, Mo and SA; Cluster II) and the east of the TMVB (Figure 1; Aj, DL, EC, NT, Ix, Am, VA, Tl, LM and CP; Cluster III) formed a gradient‐like pattern with a large contact zone west of Mexico City. The PCA further showed that genetic differentiation at this scale was weak, with each principal component explaining only a small fraction of the total genetic variance (PC1 = 6.57% and PC2 = 3.17%, Figure S2A). Individuals from Cluster I were separated along the first principal component, while samples from populations belonging to Clusters II and III were distributed along the second principal component, suggesting a pattern of isolation by distance (Figure S2). At the local scale, genetic structure and differentiation were weaker (*k* of 2; Table S2B), with only some individuals from the lower elevation stand being genetically different from the rest (Figure 1). A similar pattern was found with the PCA (Figure S2B).

Summary statistics per population are summarized in Table 1 and Table S1. At the range‐wide scale, mean expected heterozygosity (*H*_*E*_) was 0.186 and ranged from 0.091 (in SM) to 0.208 (in SA and Mo); mean observed heterozygosity (*H*_*O*_) was 0.176, and values varied between 0.073 (in SM) and 0.196 (in SA and Mo); mean nucleotide diversity (π) was 0.046 and ranged from 0.026 (SM) to 0.050 (CP). In all three cases, populations from Cluster I had significantly lower estimates than those from Clusters II and III (Table 1), suggesting lower effective population sizes and less connectivity. At the local scale, genetic diversity remained homogeneous among localities, with practically identical heterozygosity and nucleotide diversity values (mean values: *H*_*O*_ = 0.201, *H*_*E*_ = 0.207, and π = 0.046 (Table S1)).

### 3.2 Gene–Environment Association (GEA) Analyses

After multi‐collinearity tests, seven (*pH*, *Ca* ^2+^, *K* ^+^, *OC*, *EC*, *Mg* ^2+^ and *RAS*) and five (*pH*, *Mg* ^2+^, *Ca* ^2+^, *OC* and *EC*) uncorrelated chemical edaphic traits met the multivariate normality test criteria at the range‐wide (*p*‐value = 0.37) and local (*p*‐value = 0.105) scales, respectively. These variables were further used as predictors for the models below, as they all had variance inflation factors below 3 (VIF < 3).

**FIGURE 2:**
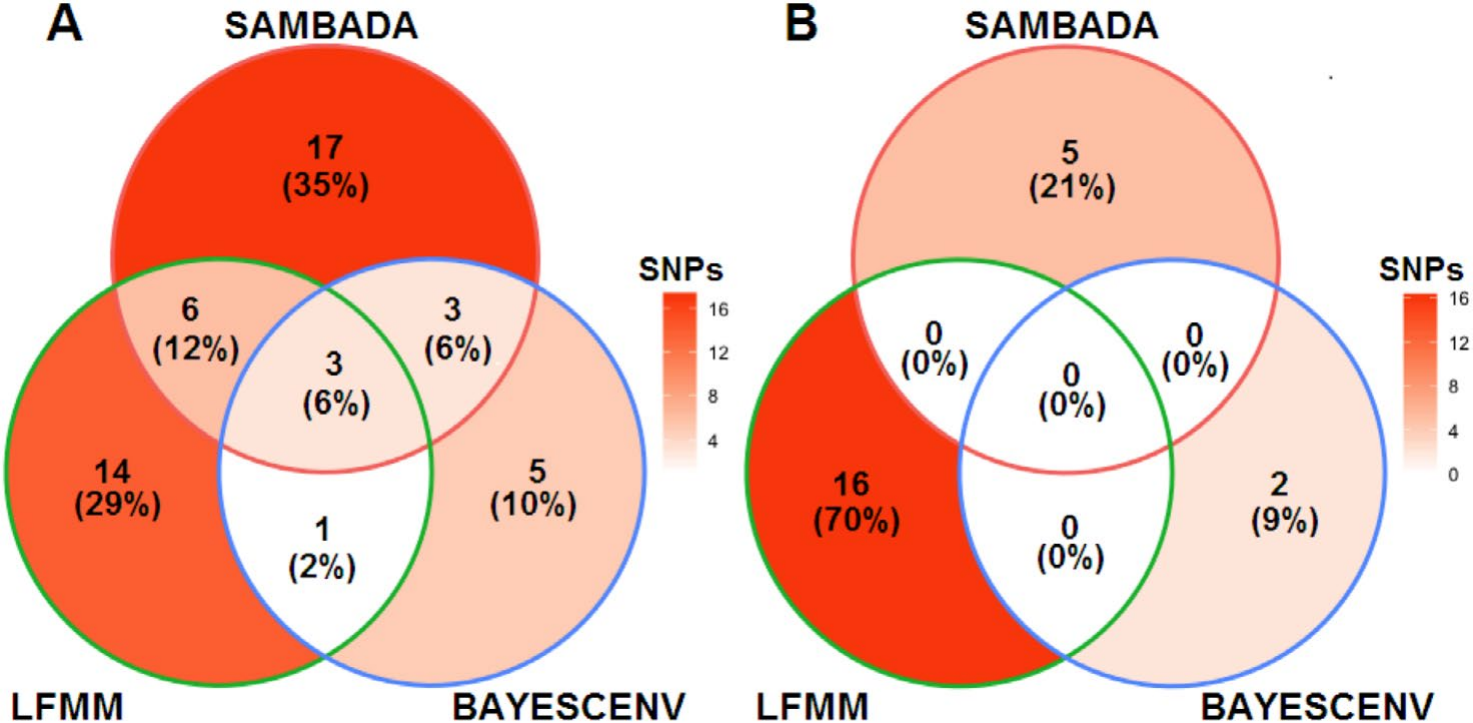
Venn diagram showing the number and percentage of candidate SNPs detected with three genotype‐environment association (GEA) methods in sacred fir (*Abies religiosa*) populations analyzed at the range‐wide (A) and local (B) scales. See Table S3 for further details.

Association analyses (LFMM, SAMβADA and BayescEnv) showed significant correlations between soil variables and 12–29 candidate SNPs at the range‐wide scale and 2–16 SNPs at the local scale (Figure 2), from which only two (*Locus371903* and *Locus636335*) overlapped between scales. At the range‐wide scale, *Ca* ^*2*+^, *pH*, and *EC* had the highest number of correlated loci (15, 10, and 7 SNPs, respectively), while *K* ^+^ and *SAR* were associated with only two and one variant, respectively. Three candidates (6.1%) were pinpointed by all three methods at this scale (*Locus23352*, *Locus24032* and *Locus296370*), while between four and nine SNPs were identified by two analyses. Only one marker (*Locus 371,068*) was associated with more than one chemical soil trait in these range‐wide analyses (*Ca* ^*2*+^ and *pH*).

At the local scale, chemical soil traits showing the highest number of correlations were EC (10) and OC (4). However, no loci were identified by more than one method, suggesting low statistical power for detecting candidates at this scale. We decided to keep all identified loci at both scales (49 and 23 respectively) and use them as response variables in the polygenic models below. It is worth noting that one of the SNPs retained at the local scale (*Locus636335*; correlated with EC) was also identified at the range‐wide level (but associated with *Ca* ^*2+*^).

Annotation was possible for nine contigs containing candidate SNPs at the range‐wide scale and for three of the loci retained at the local scale (Table 2). The most noteworthy cases included a *non‐intrinsic ABC protein 6* gene (similar to *Locus371068*; correlated with both *Ca* ^*2+*^ and pH at the range‐wide scale), a *calcium‐dependent lipid‐binding family protein* (*CaLB domain;* that contained *Locus107661*; associated with *Ca* ^*2+*^ at the range‐wide scale), and a *ribosomal protein L23/L15e family protein* (that contained *Locus636335*, which was identified at both scales).

**TABLE 2:** Putative annotation and function of contigs containing candidate single nucleotide polymorphisms (SNPs) associated with soil properties in sacred fir (*Abies religiosa*) at two different spatial scales.

Protein Names	Soil trait	Methodology			Locus	UniProt ID	Gen	Function
		L	SAM	BaE				
*Regional scale*								
Calcium‐dependent lipid‐binding (CaLB domain) family protein	*Ca* ^*+2*^		X		*Locus 107,661*	*A0A1P8BD17*	*SYTC*	*It is responsible for lipid binding and lipid transport*
Non‐intrinsic ABC protein 6	*Ca* ^*+2*^ *and pH*	X	X		*Locus 371,068*	*Q9LQK7*	*ABC17*	*Embryonic development, assembly of iron–sulphur clusters and thylakoid membrane organisation*
Nuclear RNA polymerase C2	*Ca* ^*+2*^ *and EC*	X	X		*Locus 422,516*	*A0A1P8BFS9*	*At5g45140*	*Binding to DNA and ribonucleosides, and RNA polymerase III activity*
Basic helix–loop–helix (bHLH) DNA‐binding superfamily protein	*EC*	X			*Locus 415,204*	*A0A1I9LLG6*	*At3g20640*	*DNA‐binding transcription factor activity, RNA polymerase II‐specific*
RING‐type E3 ubiquitin transferase	*Ca* ^*+2*^		X		*Locus 420,015*	*A0A5S9X0G3*	*AN1_LOCUS8581*	*Supporting ubiquitin‐dependent protein catabolic processes*
Heat shock protein DnaJ, N‐terminal with domain of unknown function	*Mg*		X		*Locus 475,342*	*D7LVR6*	*ARALYDRAFT_486177*	*Hsp70 protein binding in heat stress cases*
Pentatricopeptide repeat‐containing protein	*pH*	X			*Locus 494,058*	*D7L4E9*	*ARALYDRAFT_480422*	*Gene expression in organelles. They facilitate RNA processing, splicing, editing, stability and translation*.
DNAse I‐like superfamily protein	*Ca* ^*+2*^	X			*Locus 520,649*	*A0A1P8BDV6*	*CCR4F*	*Targeting 3′‐5’‐RNA exonuclease activity*
Ribosomal protein L23/L15e family protein	*Ca* ^*+2*^	X		X	*Locus 636,335*	*A0A178UUW8*	*At4g39880*	*Structural component of the ribosome*
*Local scale*								
Small RNA degrading nuclease 3	*EC*	X			*Locus 87,109*	*Q8RXK2*	*SDN3*	*Structural component of the ribosome and aids mRNA binding*
Pentatricopeptide repeat (PPR) superfamily protein	*OC*	X			*Locus 338,088*	*A0A1I9LQL2*	*At3g13770*	*Gene expression in organelles. They facilitate RNA processing, splicing, editing, stability, and translation*
Ribosomal protein L23/L15e family protein	*EC*	X			*Locus 636,335*	*Q9SMR5*	*T5J1750*	*Structural component of the ribosome and aids mRNA binding*

Abbreviations: *BaE*, BayeScEnv; L, LFMM; *Sam*, SAMβADA.

### 3.3 Genetic Structure Based on Candidate and Neutral Markers

The pairwise‐*F*_*ST*_ matrix at both scales showed higher genetic differentiation when using candidates than when using the non‐candidate SNPs (Table S3A,B). At the range‐wide scale, differentiation was the lowest among populations closer to the center of the species' distribution (Aj, DL, NT, Am, VT, EC, Ix and Mo), and the highest when comparing stands at the west of the species range with populations in the center or the east of its distribution. At the local scale, genetic differentiation was lower, with neutral *F*_*ST*_ values not exceeding 0.0114 (all significant; Table S3B). Although comparisons involving the lowest elevation stand (DSB) had slightly higher values than those between the two other stands.

Similar to Admixture results using the whole dataset and based on the Bayesian Information Criterion (BIC), the Discriminant Principal Component Analysis (DAPC) performed with the candidate SNPs revealed three genetic clusters at the range‐wide scale (Figure S3). At the local scale, and contrary to the Admixture results, only one genetic cluster was obtained with the retained candidates.

### 3.4 Association Between Soil Predictors and Genetic Variation

The relative contribution of environmental variables to genetic structure was inferred at both scales using distance‐based redundancy analyses (dbRDAs), for both the retained candidates (Table S4–S6 and Figure S4) and for the 20 sets of non‐candidate SNPs (i.e., non‐correlated loci matching the number and MAF of the candidates, Figure S4).

Soil predictors (constrained variance) explained a larger part of the total genetic variation than the residual variance (unconstrained) for the candidate SNPs (*p*‐value < 0.001), at both the range‐wide (17.52%) and local scales (9.67%). These same predictors only accounted for 14.42% and 6.62% of the total genetic variance of the non‐correlated SNPs at the range‐wide and local scales, respectively (*p*‐value < 0.001; Table 3). Models that included candidate loci explained conditional variance better than those that did not (13.71% and 7.94% in range‐wide and local scales, respectively). Models transferred between scales (i.e., using range‐wide candidates to predict local chemical soil variation and vice versa) were not significant. The variance explained in all simulated models was between one‐third and one‐fourth of the variance explained by the model using candidate SNPs.

**TABLE 3:** Results of partial redundancy analyses (RDA) showing the proportion of the soil chemical variance explained by polygenic models at two different spatial scales in sacred fir (*Abies religiosa*).

Source of Explained Variance (Polygenic models)	Contribution to total variance (%)		
	Inertia	Proportion	*R*_adj_^2^
Total SNPs—range‐wide scale (*n* = 1562)			
Total	22.42	100	
Conditional	0.60	2.69	
Explained	3.23	14.42	0.14
Residual	18.58	82.85	
Candidate SNPs—range‐wide scale (*n* = 49)			
Total	20.00	100	
Conditional	2.74	13.71	
Explained	3.50	17.52	0.18
Residual	13.75	68.77	
Total SNPs local scale (*n* = 1562)			
Total	17.55	100	
Conditional	0.31	1.76	
Explained	1.160	6.62	0.06
Residual	16.08	91.60	
Candidate SNPs—local scale (*n* = 23)			
Total	13.58	100	
Conditional	1.07	7.94	
Explained	1.31	9.67	0.10
Residual	11.18	82.04	

*Note:* Variance partitioning and significance values for each soil chemical variable are shown in Table S5.

Variance partitioning showed that the first two genetic eigenvalues contributed ~80% of the total variance at both scales when using candidate SNPs (F_1_ = 54.6% and F_2_ = 18.9%, range‐wide; and F_1_ = 43.6% and F_2_ = 30.5%, local), while more than four eigenvalues had to be included to account for such a portion of the total variance when using the non‐candidate loci. Interestingly, several soil chemical traits had a significant contribution to the model for explaining the candidate SNP variation at the range‐wide scale (although *Ca* ^2+^, EC, and *pH* were the most significant ones; Table S5A), while only *Ca* ^2+^, *OC*, and *EC* contributed to such model at the local scale (Table S5B).

**FIGURE 3:**
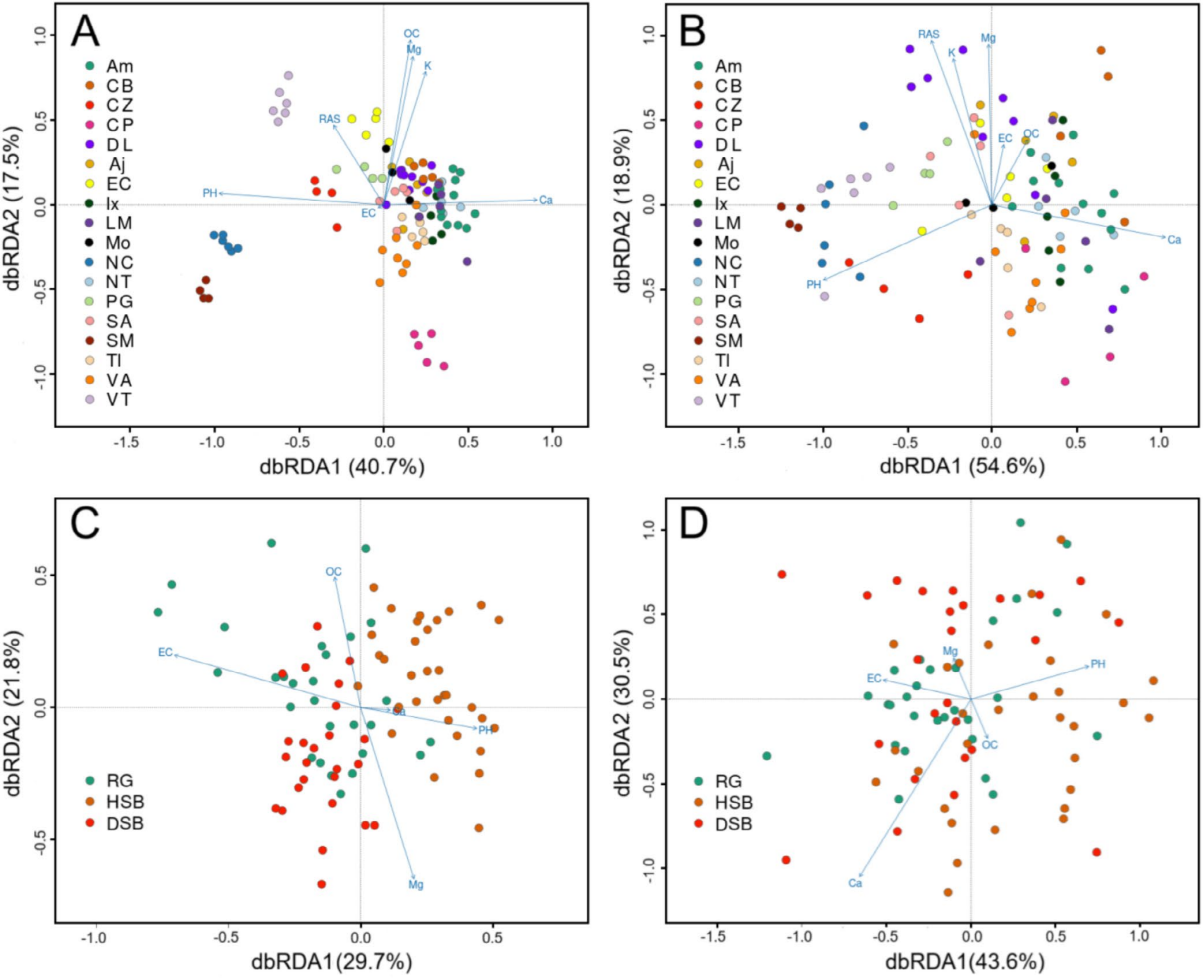
Redundancy analyses (RDA) biplots showing soil traits and SNP co‐variation in sacred fir (*Abies religiosa*) populations analyzed at two different spatial scales. Soil variables as blue arrows, and populations as colored circles. Left (A, C) analyses performed using the total SNP dataset. Right (B, D) analyses carried out with retained candidate SNPs (see text Figure 2 and Table S3). Top (A, B) co‐variation at the range‐wide scale. Bottom (C, D) co‐variation at the local scale (see Table 1 and Table S1 for population locations and abbreviations).

dbRDAs further showed that populations could be better differentiated at both scales when using neutral markers than candidate SNPs (Table S6), although with low correlation values (*R*_*adj*_^*2*^ = 0.14 and 0.06 at the range‐wide and local scales, respectively; Table 3 and Figure 3). These analyses imply that chemical edaphic traits are differentially influencing the distribution of candidate and neutral SNPs between scales, except for *OC* and *EC*, which were both significant in all cases.

For the validation of polygenetic models using random numbers of neutral SNPs that matched the number and MAF of the candidates (49 and 23 for range‐wide and local scales, respectively), we were only able to explain a very low portion of the variance in all cases. In addition, reduced proportions of the variance were explained when using the range‐wide scale outliers to calculate the variance constrained at the local scale and vice versa (Figure S4).

### 3.5 Spatial Distribution of Putatively Adaptive Genetic Variation

**FIGURE 4:**
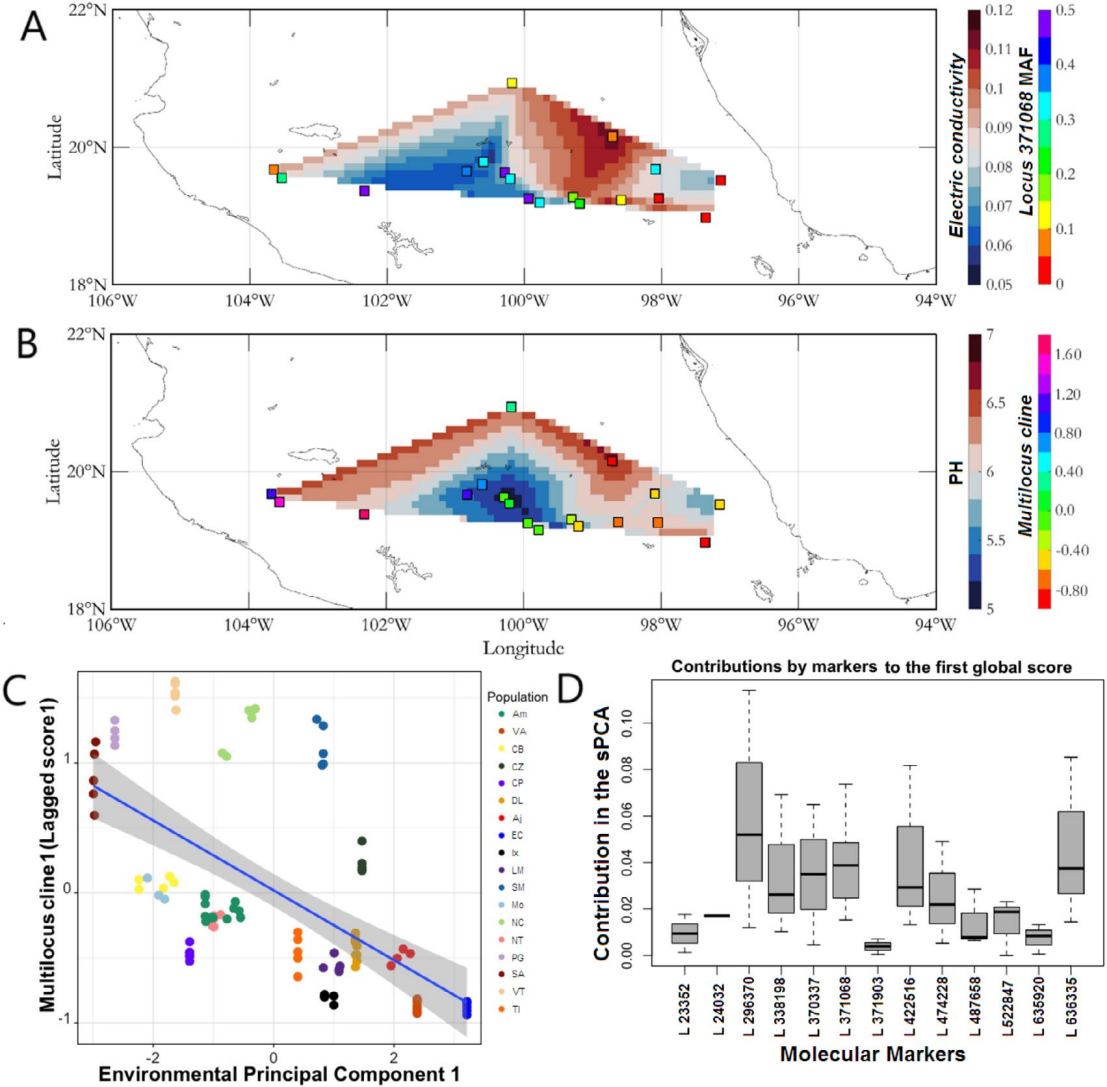
Spatial distribution of soil electric conductivity (EC; A), and pH (B) variation in central Mexico and correlation with allele frequency at *Locus 371,068* (A) and population *lagged scores* of a multi‐locus cline composed of 49 candidate SNPs in sacred fir (*Abies religiosa*) (B). *Lagged scores* were obtained from a sPCA analysis, and reflect the genetic variability linked to the spatial distance between sites. (C) Linear adjustment between the first PCA‐axis summarizing soil variability in central Mexico and the *lagged scores* of each individual in the multi‐locus cline. Diagonal represents the best‐fit regression lines (*R* ^*2*^_*adj*_ = 0.31; *p‐value* < 0.05). (D) Boxplot showing the contribution of the top 13 markers to the multi‐locus cline of the sPCA analysis.

To evaluate the role of geography in the distribution of candidate SNP variation at both scales, we used spatial Principal Component Analyses (sPCA; Figure 4, Figures S5 and S6). We extracted two *lagged scores* (multi‐locus clines) that summarized the genetic variability of the candidate SNPs linked to the spatial distribution of populations (range‐wide scale) or individuals (local scale). We then performed multiple stepwise linear regressions between these clines and the edaphic predictors used for the GEA analyses, both for each individual chemical soil trait and after summarizing chemical soil variation in two principal components (EPCs 1 and 2; Table 4 and Table S7).

**TABLE 4:** Results of stepwise multiple regression analyses between the distribution of spatial genetic variation (*lagged scored* 1 and *lagged score* 2) and range‐wide soil chemical variation in *A. religiosa* .

Equation Linear Model y = b_0_ + b_1_x_1_	*R*_adj_^2^	*p*‐value
Lagged scores 1		
0.0243 − 0.367 × *Mg* ^2+^	0.198	1.6 × 10^−6^
0.0238 + 0.067 × *K* ^+^	—	> 0.05
0.0236 − 0.594 × *Ca* ^2+^	0.401	2.2 × 10^−14^
0.0241 − 0.168 × *pH*	0.122	0.0038
0.0248 + 0.224 × *OC*	0.069	0.0044
0.0249 − 0.441 × *EC*	0.31	2.0 × 10^−9^
0.0229 + 0.195 × *SAR*	—	> 0.05
0.0199 − 0.268 × *EPC1*	0.339	7.8 × 10^−11^
0.0239 + 0.058 × *EPC2*	—	> 0.05
0.024 − 0.144 × *EC* − 0.465 × *Ca* ^2+^	0.438	2.2 × 10^−16^
Lagged scores 2		
0.0019 − 0.195 × *Mg* ^2+^	0.185	3.7 × 10^−6^
0.0017 + 0.018 × *K* ^+^	—	> 0.05
0.0017 − 0.080 × *Ca* ^2+^	—	> 0.05
0.0025 − 0.305 × *pH*	0.451	3.7 × 10^−16^
0.0248 + 0.224 × *OC*	0.237	1.2 × 10^−7^
0.0023 − 0.245 × *EC*	0.304	1.1 × 10^−9^
0.0017 + 0.011 × *SAR*	—	> 0.05
−0.0009 − 0.154 × *EPC1*	0.373	5.5 × 10^−12^
0.0018 − 0.060 × *EPC2*	—	> 0.05
0.003–0.128 × *EC* − 0.244 × *pH*	0.523	2.1 × 10^−16^
0.003–0.114 × *EC* − 0.214 × *pH* + 0.08 × *OC*	0.548	2.3 × 10^−16^

Abbreviations: EC, electric conductivity; EPC, environmental principal component; OC, organic carbon; SAR, sodium absorption ratio.

At the range‐wide scale, significant correlations were observed for both *lagged scores* (*1 and 2*) and EPC1 (*R*_*adj*_^*2*^ = 0.34–0.38, *p*‐value < 0.001; Figure 4, Figure S6 and Table S7), indicating a significant contribution of geographic proximity to both soil and candidate SNP variation. When chemical soil variables were analyzed individually, the most significant correlations for *lagged score 1* were with *EC* and *Ca* ^*2+*^ (*R*_*adj*_^*2*^ = 0.31 and *R*_*adj*_^*2*^ = 0.40, respectively); for *lagged score* 2, significant correlations were observed for *pH*, *EC*, and *OC* (*R*_*adj*_^*2*^ = 0.45, 0.304 and 0.24, respectively) (Table 4).

At the local scale, only *lagged score 1* was correlated with EPC1, while *lagged score 2* showed no correlations. The only individual variables that were correlated with both *lagged scores* at this local scale were *EC* (*R*_*adj*_^*2*^ = 0.155, *p*‐value < 0.001) and *OC* (*R*_*adj*_^*2*^ = 0.082, *p*‐value = 0.01) (Table S7).

Candidate SNPs contributed differentially to the inferred multi‐locus clines at the range‐wide scale (Figure 4, bottom right), with those SNPs that were detected by more than one method (*Locus 296,370*, *Locus 371,068*, *Locus 370,337*, *Locus 422,516 and Locus 636,335*) having a larger contribution than those detected by only one method. It is worth noting that one of these loci (*Locus 636,335*) also had a strong contribution to the multi‐locus cline at the local scale (Table 2). Among the candidates detected at the range‐wide scale, loci *296,370*, *370,737*, and 3*71,068* had contrasting genotype compositions in soils differing in *Ca* ^*2+*^, *EC*, and *pH*. For instance, soils with higher *Ca* ^*2+*^ concentrations and higher *EC* and *pH* tended to have AA and AT genotypes at *Locus 296,370* and GG genotypes at loci *370,737* and *371,068* (Figure S7). At the local scale, this was inferred for loci *337,921* and *636,335* and soil *EC* and *OC*, but with much less statistical power (Figure S8).

## 4 Discussion

We explored whether the same gene‐edaphic associations could be detected at two different spatial scales (range‐wide and local) in a long‐lived tree from central Mexico (sacred fir; *Abies religiosa*). Our findings revealed a complex genomic architecture that differs between scales, suggesting an intricate interplay between chemical soil factors and the distribution of genetic diversity. They further imply that adaptation to soil has a polygenic basis, likely influenced by the local genetic composition and the degree of population isolation (Figure 2 and Table 3). Researchers focusing on forest tree adaptation should start addressing chemical edaphic variation and investigating the genetic architecture underlying soil‐related selective pressures in common garden experiments; they should also start taking edaphic traits into account for bonifying management and conservation programs. It is likely that similar results will be observed in other forest trees.

### 4.1 Genetic Structure and Gene–Environment Associations

We obtained the same phylogeographic structure previously reported for *A. religiosa* , involving one isolated genetic pool in the westernmost portion of the Trans‐Mexican Volcanic Belt and a gradient of differentiation between eastern and central populations (Cruz‐Nicolás et al. 2019; Giles‐Pérez et al. 2022). As discussed in previous phylogeographic and population genetics works, the lower genetic diversity observed in the western populations could be associated with orogenetic processes that isolated them and diminished both their effective population sizes and their capacity to accumulate genetic diversity (Cruz‐Nicolás et al. 2020; Giles‐Pérez et al. 2022). In contrast, the high amounts of genetic diversity observed in central and eastern populations reflect larger historical effective population sizes and population connectivity, probably because of a greater environmental and population stability (Cruz‐Nicolás et al. 2019, 2020; Giles‐Pérez et al. 2022).

As expected for conifers, genetic structure was subtle at the local scale, indicating rampant homogenization through pollen‐mediated gene flow (Figure 1; Heredia‐Bobadilla 2012; Méndez‐González et al. 2017; Ortiz‐Bibian et al. 2017).

dbRDA analyses further suggested that this subtle differentiation was aligned with soil dissimilarities, especially along the first discriminant axis, which was loaded by soil electric conductivity (*EC*) and the amount of organic carbon (*OC*). Chemical edaphic factors were also important for explaining range‐wide scale differentiation, again including *EC* and *OC*, but most importantly, traits like the *Ca* ^*2+*^ concentration and soil *pH* (Figure 3a). Such an interplay between the local soil chemical properties and the population genetic structure is a recurrent factor in plant landscape genetic studies, which have often shown that edaphic constraints are important drivers of local adaptation (Bragg et al. 2015; Méndez‐González et al. 2017; Orsini et al. 2013).

Up to 70 SNPs showed associations with chemical soil traits; 12–29 candidates were observed at the range‐wide scale, while 2–16 SNPs were detected at the local scale (Figure 2). Together, these SNPs explained larger portions of the total chemical soil variance at both scales than random SNP sets matching their number and MAF. Although our study is likely underpowered to pinpoint most of the variants contributing to soil adaptation (because of the low number of individuals surveyed and a suboptimal coverage of the ~17 Gb genome of *A. religiosa* ), it is noteworthy that associations were recurrently inferred with conceptually different statistical methods and generally involved the same chemical soil traits (i.e., *pH*, *Ca* ^*2+*^, and *EC*; Figure 2; Table 2). Even though there was little overlap between the candidates detected at both scales (only two out of 70 SNPs), the annotation of the contigs that contained such candidates indicated that some of them could be more than mere false positives. Indeed, many candidates were located within genes involved in the regulation of gene expression and membrane transport, including a contig that carried a SNP associated with *Ca* ^*2+*^, which was annotated as a *calcium‐dependent protein* (see below; Table 2).

To understand how a species responds to putatively similar selective pressures at different geographic scales, we first need to consider the evolutionary history of populations, their genetic makeup, and the likelihood of individuals to locally adapt (Zellmer and Knowles 2009; Capblancq et al. 2020). For instance, the main genetic lineages of *A. religiosa* started diverging ~1.0 Ma ago (Giles‐Pérez et al. 2022), and while the western lineage remained effectively isolated after its initial divergence, the central and eastern genetic pools exchanged genes during the last ~200Ky (Giles‐Pérez et al. 2022). This demographic history likely produced the unique genetic associations observed herein between the western and central/eastern genetic pools, despite having common chemical edaphic pressures, including *pH*, *Ca* ^*2+*^ and *EC* variation (Figures 3 and 4). The demographic history of *A. religiosa* further hints at an eventual, and recent, genetic exchange of adaptive alleles between the eastern and central lineages (Giles‐Pérez et al. 2022). However, exploring this would necessitate a different sampling and analytical approach than the one used here (Giles‐Pérez in prep.).

While the range‐wide distribution of genetic variation in sacred fir is likely driven by the interaction between demographic history and chemical edaphic adaptation, the distribution of within‐population genetic variation is apparently modeled by traits that can be viewed as proxies of soil cation exchange capacity (i.e., *EC* and *OC*), a key property that influences ion retention, nutrient availability, and the structural stability of the substrate (Li et al. 2015; Madritch et al. 2006; Terés et al. 2019). This indicates that tree growth and physiological performance could be at the base of local‐scale edaphic adaptation, such as observed in a previous study in the same stands surveyed herein (Arenas et al. 2021). In addition, the implication of *EC* and *OC* in adaptation may also be hinting that the role of the decomposing microorganisms that make nutrients available for plant uptake might be at stake (Raven and Andrews 2010; Schweitzer et al. 2011). The quantity and composition of microorganisms in the soil can indeed vary greatly at very short distances (Kubota et al., 2015; Purahong et al. 2016; Chen et al. 2019) and should be the focus of future studies aiming to understand edaphic tree adaptation at the local scale.

It may be, however, argued that local‐scale adaptation in sacred fir is actually driven by soil traits that were not considered in the present study, such as ammonium concentration (*NH*_*4*_; Arenas et al. 2021), bulk density (Argüelles‐Moyao and Garibay‐Orijel 2018), water retention capacity, relative humidity (Csilléry et al. 2020) or soil aridity (Steane et al. 2017). Disentangling the role of the many variables involved in soil variation, most of which are expected to be correlated, is indeed a complex task that will need experiments with controlled soil conditions, and which are out of the scope of the present study. Furthermore, GEA studies combining climate and soil traits are also necessary for exploring how they intertwine to affect water availability and local adaptation, as proposed for other forest trees (Csilléry et al. 2020; Zimmermann et al. 2025).

### 4.2 Edaphic Adaptation Has a Polygenic Architecture in *Abies religiosa*

We used multilocus models based on candidate SNPs to provide a first approximation to polygenic soil adaptation in a forest trees, which allowed us to explain 16.96 and 23.95% of the edaphic variance at the local (23 SNPs) and range‐wide (49 SNPs) scales, respectively (Table 3). This mirrors the amount of variance explained by recent polygenic models for climate adaptation and pest resistance in other forest trees (e.g., De La Torre, Sekhwal, and Neale (2021); De La Torre, Wilhite, et al. (2021); Neophytou et al. (2022)).

In a polygenic a model, it is the locus combinations, rather than specific polymorphisms at genes with major phenotypic effects, that are driving local adaptation (Savolainen et al. 2007; De La Torre et al. 2014; Carvalho et al., 2021; George et al. 2021). In other words, polygenic adaptation relies on multiple (and often redundant) loci of small effect, upon which moderate selective pressures operate, generating subtle allele frequency changes among populations (Crouch and Bodmer 2020). This implies that different genomic backgrounds (i.e., genetic pools) may harbor non‐overlapping gene–environment associations when submitted to similar selective pressures (Prunier and Verta 2016; Chen et al. 2021; George et al. 2021).

In the case of soil adaptation, one would expect that a polygenic basis will allow for a flexible and robust response to environmental changes operating at various levels, while still maximizing nutrient uptake (Baxter et al. 2010; Kellermeier et al. 2013).

Such a response should involve several phenotypic traits. For instance, root architecture (i.e., root length, growth and branching patterns, and hair density), which secures nutrient supply, and plant anchorage and support (Maurel and Nacry 2020), or the presence of mycorrhizae, which enhances nutrient uptake and plant resistance to drought (Revillini et al. 2016). However, the genomic architecture of virtually all the phenotypic traits that could be involved in soil adaptation is unknown, especially because of the intrinsic difficulties of root phenotyping. Indeed, the functional genomics of root architecture has only been explored in model plants, like *Arabidopsis* or maize (e.g., Iyer‐Pascuzzi et al. 2009; Hochholdinger et al. 2018), and the composition of mycorrhizal communities has only begun to be recurrently investigated with the onset of metabarcoding and metagenomics (e.g., Argüelles‐Moyao and Garibay‐Orijel 2018). Thus, we urge researchers to combine the study of edaphic adaptation with root phenomics and mycorrhizae metabarcoding, and consider the interaction between tree genotype, soil microbiota, and nutrient availability. Such a combination will surely translate into better forest management, conservation, and assisted migration plans (Li et al. 2022; Roux et al. 2023).

### 4.3 The Putative Role of Chemical Soil Properties in Fir Adaptation

The soil traits that were recurrently associated with genomic variation at the range‐wide level in *A. religiosa* (i.e., *pH*, *EC*, and *Ca* ^2+^; Table 2; Figure 4A; and Table S5) could be indicative of population differentiation in cation exchange capacity, nutrient fixation, and response to soil acidity (Zancarini et al. 2012; Xue et al. 2017). Indeed, the complex relationship between *pH* and EC (and thus cation exchange capacity) is here evidenced through an edaphic differentiation gradient, with the south‐central sacred fir populations growing on more acidic soils than the eastern and western stands (Figure 4). *pH* is known to directly affect nitrogen and phosphorus availability (Lu et al. 2012), and it also modifies the solubility of nutrients that are necessary for root physiological activities (i.e., *Al*, *Fe* ^2+^, *Mg* ^*2*+^, and *K* ^+^; Cakmak 2013; Wang et al. 2013), but which are toxic when in excess (Wu et al. 2019; Zhang et al. 2018).

*Ca* ^2+^ also seems to be a major driver of edaphic adaptation in this species. This ion is essential for cell reproduction and root development, and it also limits mycorrhizal formation, which may affect nutrient uptake and tree survival (Lehto and Grace 1994; Børja and Nilsen 2009). The distribution of tropical ectomycorrhizal trees is, however, independent of the soil chemical composition (Medina‐Vega et al. 2024), and sacred fir populations from central Mexico (all from our genetic cluster III) apparently do not differ in their mycorrhizal composition (Argüelles‐Moyao and Garibay‐Orijel 2018). It is thus necessary to expand mycorrhiza studies to other *A. religiosa* stands, particularly those from other genetic clusters, which have particularly low *Ca* ^2+^ concentration (e. g. cluster I). It is also necessary to start developing common garden or reciprocal transfer studies to explore the interaction between genetic composition and edaphic variation. This will not only help to elucidate the edaphic adaptive mechanisms but will also be key for developing effective forest conservation strategies, including assisted migration, in the context of current environmental degradation (Argüelles‐Moyao and Garibay‐Orijel 2018).

### 4.4 Functional Annotation of Soil‐Associated Genes

The genes identified in the present study had little overlap between methods and likely represent a small subsample of those that may be involved in edaphic adaptation in firs (not to mention that some of them may be false positives). However, they do show common trends, both in the traits they are associated with and the annotation we were able to perform. Such annotations mostly included genes involved in gene regulation, signaling, and stress response.

Some notable examples include *Locus636335*, which contained a SNP that was detected at both the range‐wide and local scales, and loci *422,516* and *371,068*, both of which harbored variants that were correlated with more than one edaphic trait at the range‐wide scale (Table 2). The first locus is similar to a *L23/L15e* family ribosomal protein, whose members are involved in translation activities and have been associated with plant growth and flowering (Dai et al. 2017). The second locus, *422,516*, is similar to a nuclear *RNA* polymerase *C2* gene. Genes within this family are involved in gene expression activities, particularly transcription termination (Lin et al. 2004) and have been associated with seed formation and drought resistance in angiosperms (e.g., Li et al. 2019; Thiruppathi 2020). The third locus, *371,068*, was annotated as a non‐intrinsic *ABC* protein 6. This large family of protein transporters is involved in various homeostatic processes, including metal detoxification and nutrient movement (Kang et al. 2011).

It is worth noting that SNPs within the above‐mentioned genes, together with those from three more contigs, were associated with *Ca* ^*2+*^ (Table 2). Two of these additional contigs showed similarities with genes involved in gene regulation (i.e., loci *420,015* was annotated as a ubiquitin transferase, and *520,649* as a *DNAse I*‐like gene). The third locus, *Locus107661*, is perhaps the most noticeable, as it was annotated as a calcium‐dependent lipid‐binding family protein (*CaLB* domain). These proteins have a fundamental role in signaling and response to stress (Tuteja and Mahajan 2007; Kim et al. 2009; Atif et al. 2019), some of which are strongly upregulated by low temperature in *Picea* and *Populus* (Holliday et al. 2008; Estravis‐Barcala et al. 2020). They include calcium‐dependent protein kinases (CDPKs), which are further involved in drought and cold tolerance in various plants (Chen et al. 2013; Xiao et al. 2022). Genes containing *CaLB* domains are thus good candidates for understanding how calcium signaling is involved in edaphic adaptation in firs and other plants.

Finally, two candidate loci were annotated as members of the pentatricopeptide repeat (*PPR*) protein superfamily, *494,058* at the range‐wide scale, and *338,088* at the local scale (Table 2). Members of this family are known to play a key role in abiotic stress response, particularly drought and salinity (Miranda et al. 2018). These proteins further participate in RNA metabolism and are involved in the protection of membrane integrity and ion transport (Barkan and Small 2014); all of which are functions that could be related to edaphic adaptation in plants. Additional studies with controlled conditions are thus necessary to prove causality and show that these genes are also playing similar roles in conifers (including *A. religiosa* ).

## 5 Conclusions and Perspectives

Edaphic pressures operating at various geographic scales are main drivers of local adaptation in sacred fir (*Abies religiosa*). Soil chemical variation should be thus integrated into forest management and biological conservation strategies for this, and probably most forest tree species. Genetic clusters and soil variables identified in this study can indeed inform current management programs. For instance, assisted migration strategies, such as those proposed by Sáenz‐Romero et al. (2024), could be refined if they include the soil chemical properties of the receiving localities.

Future studies could also build on the hypotheses discussed here; for instance, by focusing on root phenomics and metagenomic analyses of both soil microbial communities and mycorrhiza (Hirte et al. 2018; Zhou et al. 2023). Root phenomics could be facilitated by 3D imaging and modeling techniques (i.e., Takahashi and Pradal 2021), which could help disentangle the different aspects that contribute to root architecture (Li et al. 2022). This could be complemented by functional genomic analyses in seedlings, as has been performed in *Arabidopsis* and maize (Li et al. 2019; Thiruppathi 2020) or in combination with rooted cuttings (e.g., Rioux et al. 2007). The use of these techniques may further help select tree genotypes that are better adapted to specific soil and climate conditions (Ritchie 1991; Koskela et al. 2014). This genotype selection could also be based on advanced sequencing technologies and machine learning analysis that incorporate highly polygenic models and include various components involved in edaphic adaptation (and its interaction with climate). Finally, soil traits and mycorrhiza composition should play a capital role in conservation plans, as demonstrated in previous transplant experiments in *A. religiosa* (Ortiz‐Bibian et al. 2017; Argüelles‐Moyao and Galicia 2024). However, while sacred fir is a good model to perform such studies because of its distribution in heterogeneous edaphic landscapes, tree species with more developed genomic tools and common garden experiments must also be considered to perform such experiments (Plomion et al. 2016; Guerrero et al. 2018).

## Conflicts of Interest

The authors declare no conflicts of interest.

## Supporting information


Data S1.

## Data Availability

Data from this study are available at BioProject accession https://figshare.com/s/36e5eda4aff0f43e9f41 at the figshare database.
