# Supplementary material for: Contribution of Range‐Wide and Short‐Scale Chemical Soil Variation to Local Adaptation in a Tropical Montane Forest Tree
Source: Evol Appl. 2025 Jul 9;18(7):e70116. doi: 10.1111/eva.70116 (PMC12241699; doi:10.1111/eva.70116)
Supplement: Supplementary file 1 — Data S1. [file EVA-18-e70116-s001.docx]

**Contribution of range-wide and short-scale chemical soil variation to local adaptation in a tropical montane forest tree**

**SUPPLEMENTARY MATHERIAL**


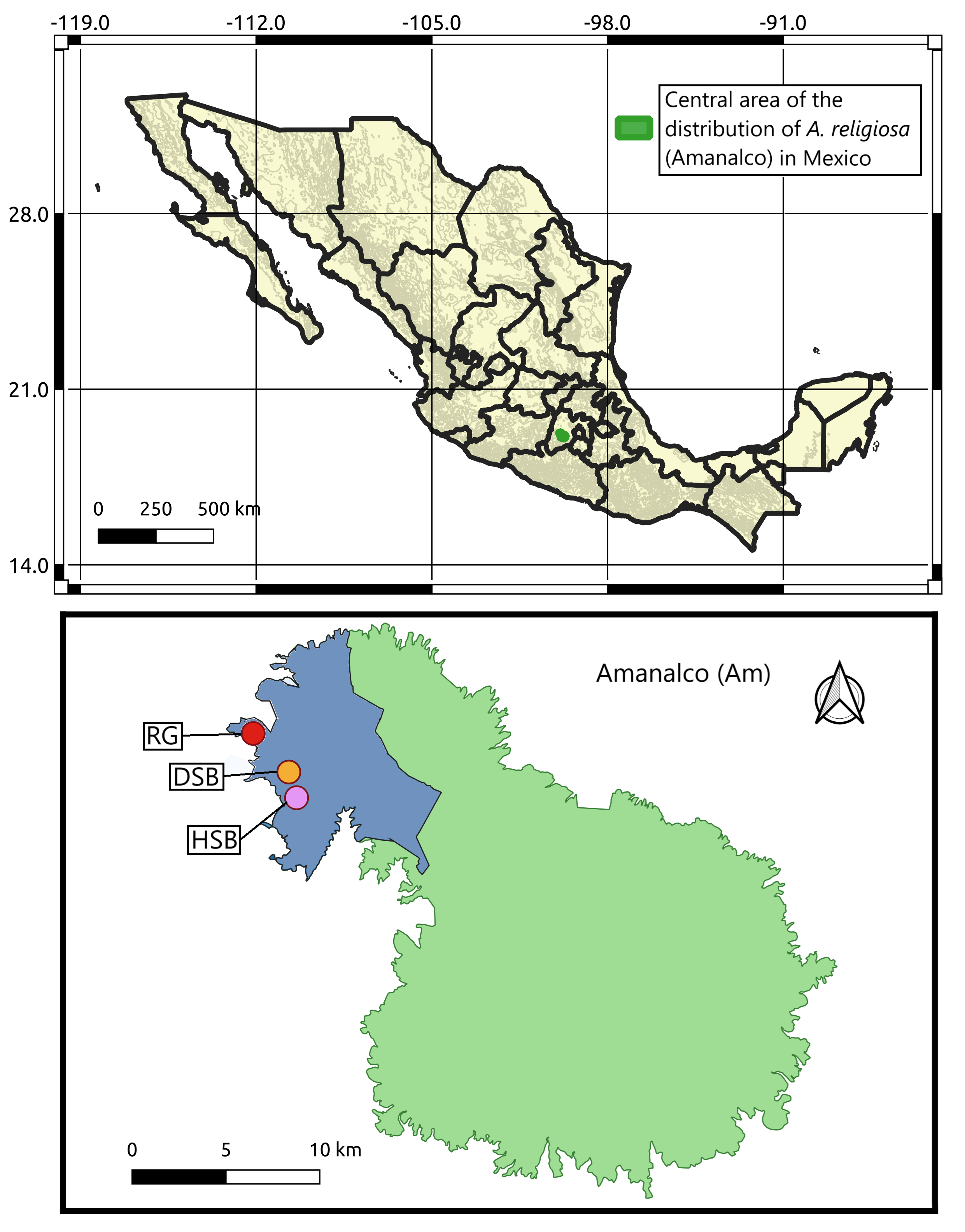


**Suppl. Fig. 1.** Map showing the location of the Nevado de Toluca flora and fauna protection area (NTFFPA) in Mexico (Top), and of the municipality of Amanalco within that protected area (bottom). Populations that were sampled for studying the local-scale variation of *Abies religiosa* are shown as colored dots. Rincón de Guadalupe (RG, red) and Down San Bartolo (DSB, orange) and High San Bartolo (HSB, pink) as well.


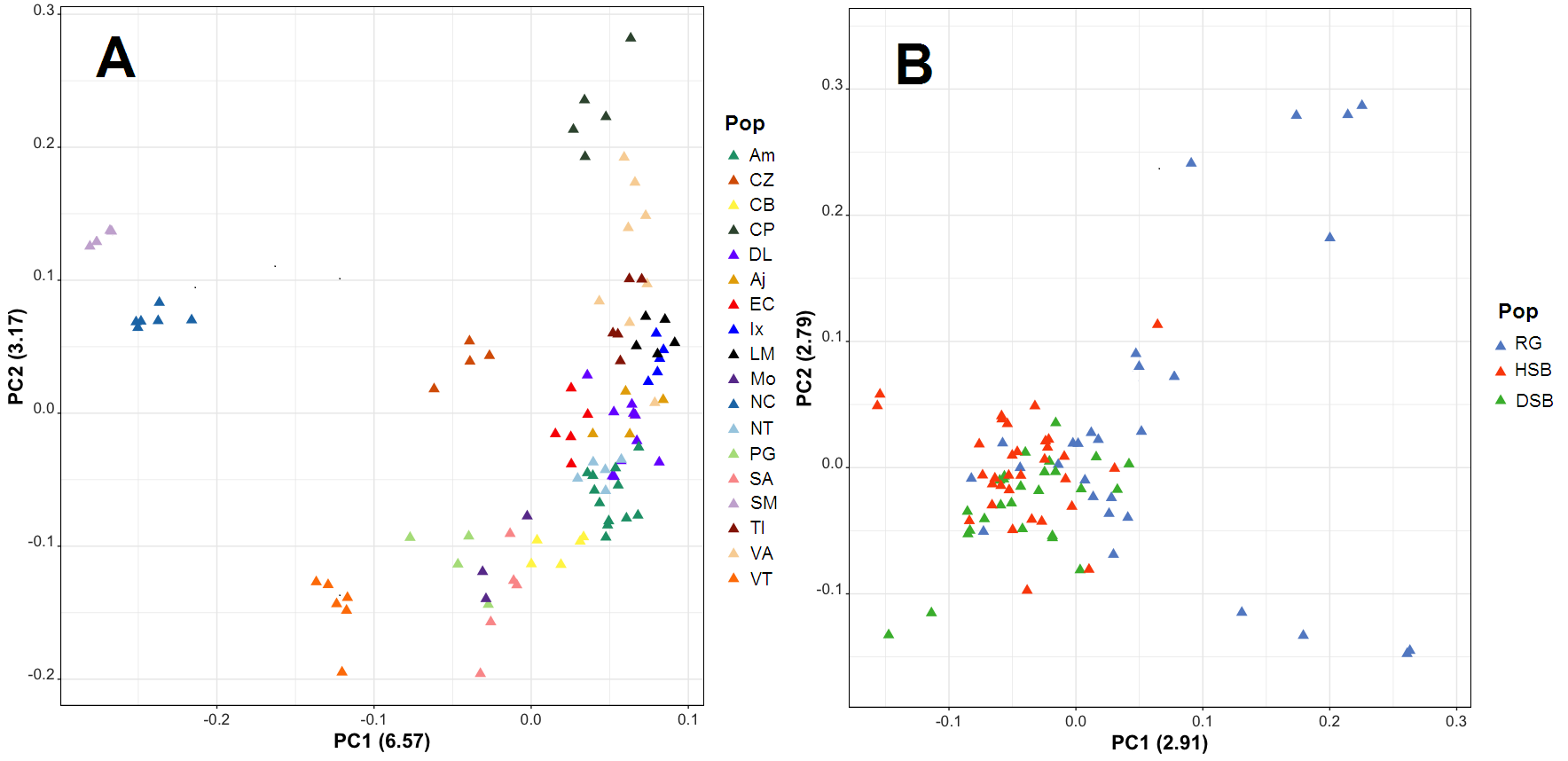


**Suppl. Fig. 2** Unsupervised grouping of individual genotypes (from 1,585 SNPs) for sacred fir (*Abies Religiosa*) populations analyzed at the range-wide scale along the Tran-Mexican Volcanic Belt in central Mexico **(A)** and at the local scale **(B)**. Numbers in parentheses next axes names indicate the percentage of explained variance. See Fig. 1 for population locations. Abbreviations **Suppl. Fig. 2.A**: Am, Amanalco; CZ, Cerro Zamorano; CB, Cerro Blanco; CP, Cofre del Perote; DL, Desierto de Leones; Aj, Ajusco; EC, El Chico; Ix, Ixtapalucan; LM, La Malinche; M, Monarca; NC, Nevado de Colima; NT, Nevado de Toluca; PG, Puerta Garnica; SA, San Andrés; SM, Sierra Manantlán; Tl, Tlaxco; VA, Volcán Atlitzín; VT, Volcán Tancítaro. **Suppl. Fig. 2.B** RG, Rincón de Guadalupe; HSB, Hight San Bartolo; DSB, Down San Bartolo.


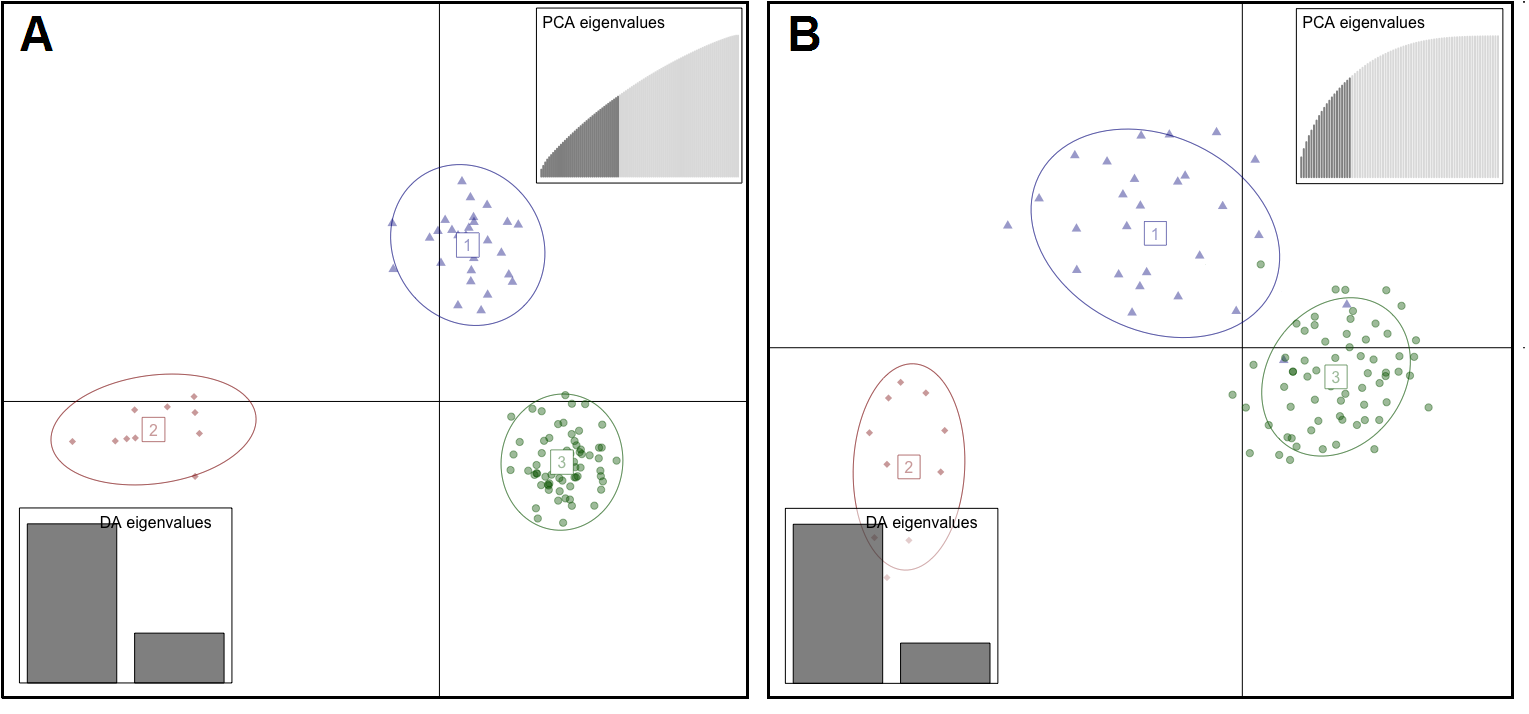


**Suppl. Fig. 3** Population genetic structure of 102 individuals from 18 sacred fir (*Abies religiosa*) populations using (**A**) 49 candidate and (b) 1,536 putatively neutral SNPs.


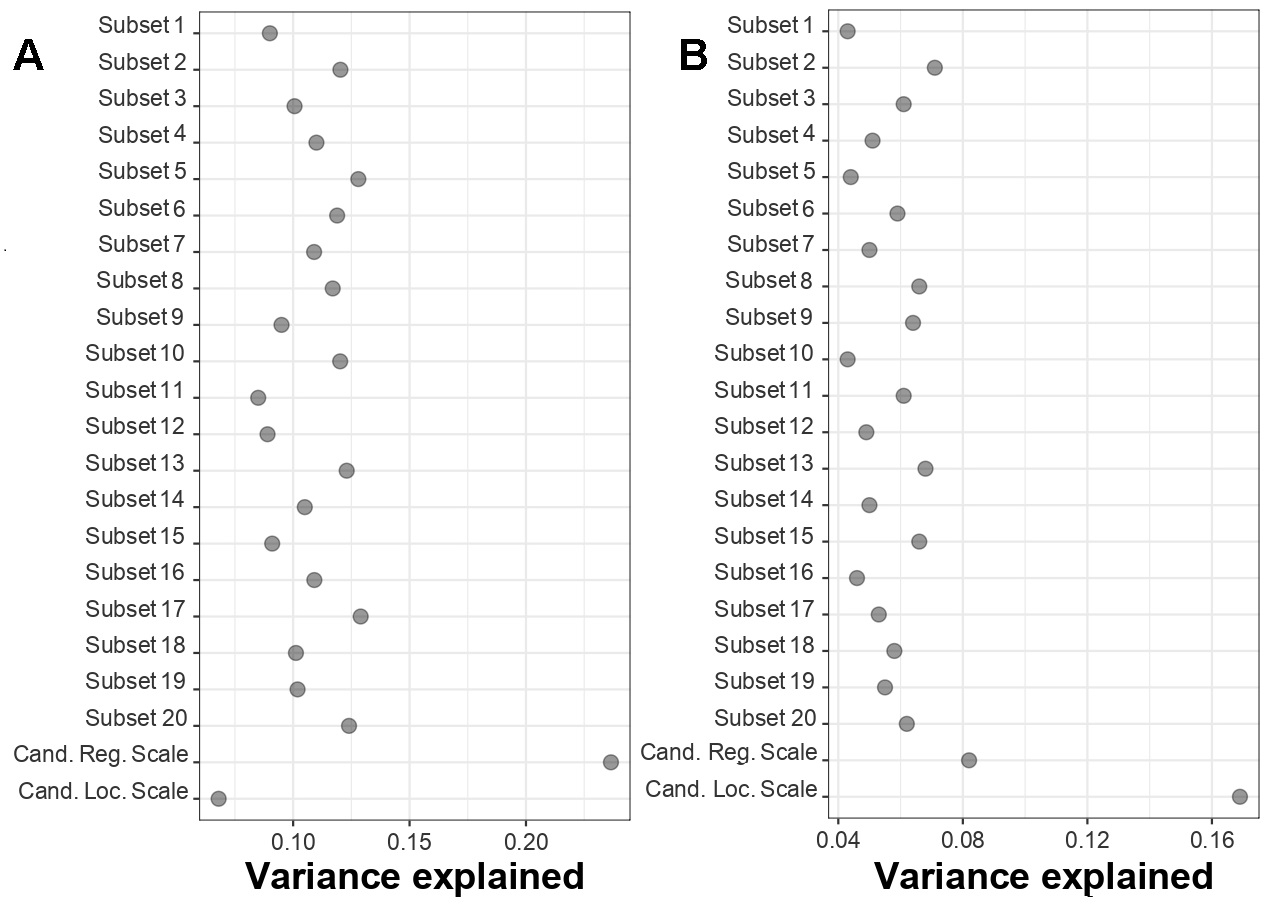


**Suppl. Fig. 4** Percentage of the variance explained by different polygenic models built with redundancy analyses (RDA) using subsets of putatively neutral loci (subsets 1-20) and retained candidates (Cand.) following genotype environment associations (GEA) in sacred fir populations surveyed at the range-wide **(A)** and local scales **(B)**. Scatterplots show the relative contribution obtained for each RDA.


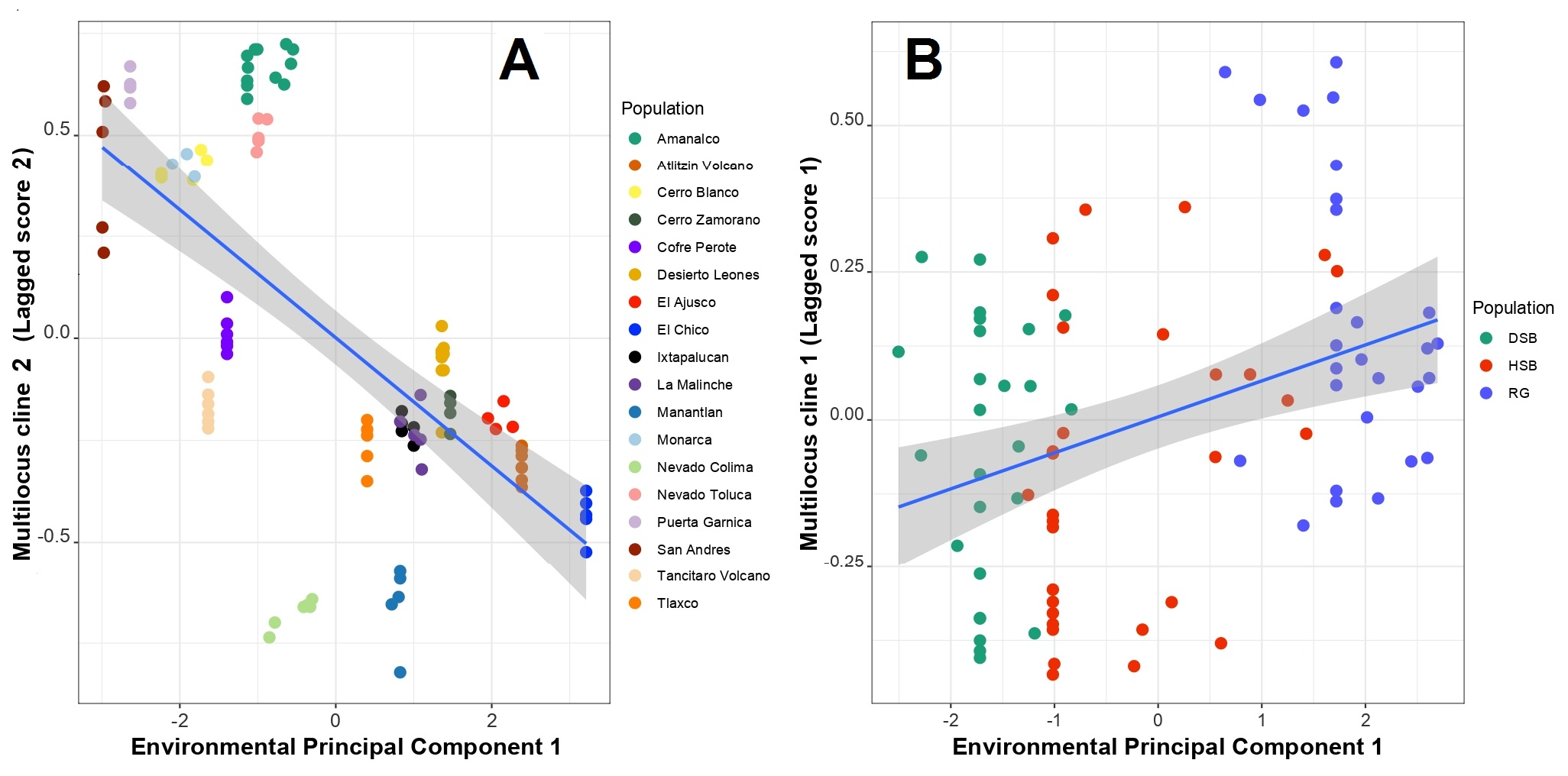


**Suppl. Fig. 5** Correlation between the first environmental (chemical soil) principal component (*x-*axis) and genetic multilocus cline 2 (*lagged score* 2, *y-*axis) at the range-wide scale **(A)** and the first environmental (chemical soil) principal component 1 (*x-*axis) and multilocus cline 1 (*lagged score* 1, *y-*axis) at the local scale **(B)** for sacred firs (*Abies religiosa*) populations in central Mexico. The diagonal line represents the best-fit regression lines (*R^2^_adj_* = 0.37; *P* - value = 5.5 × 10^-12^ and *R^2^_adj_* = 0.12; *P*-value = 6.0 × 10^-4^).


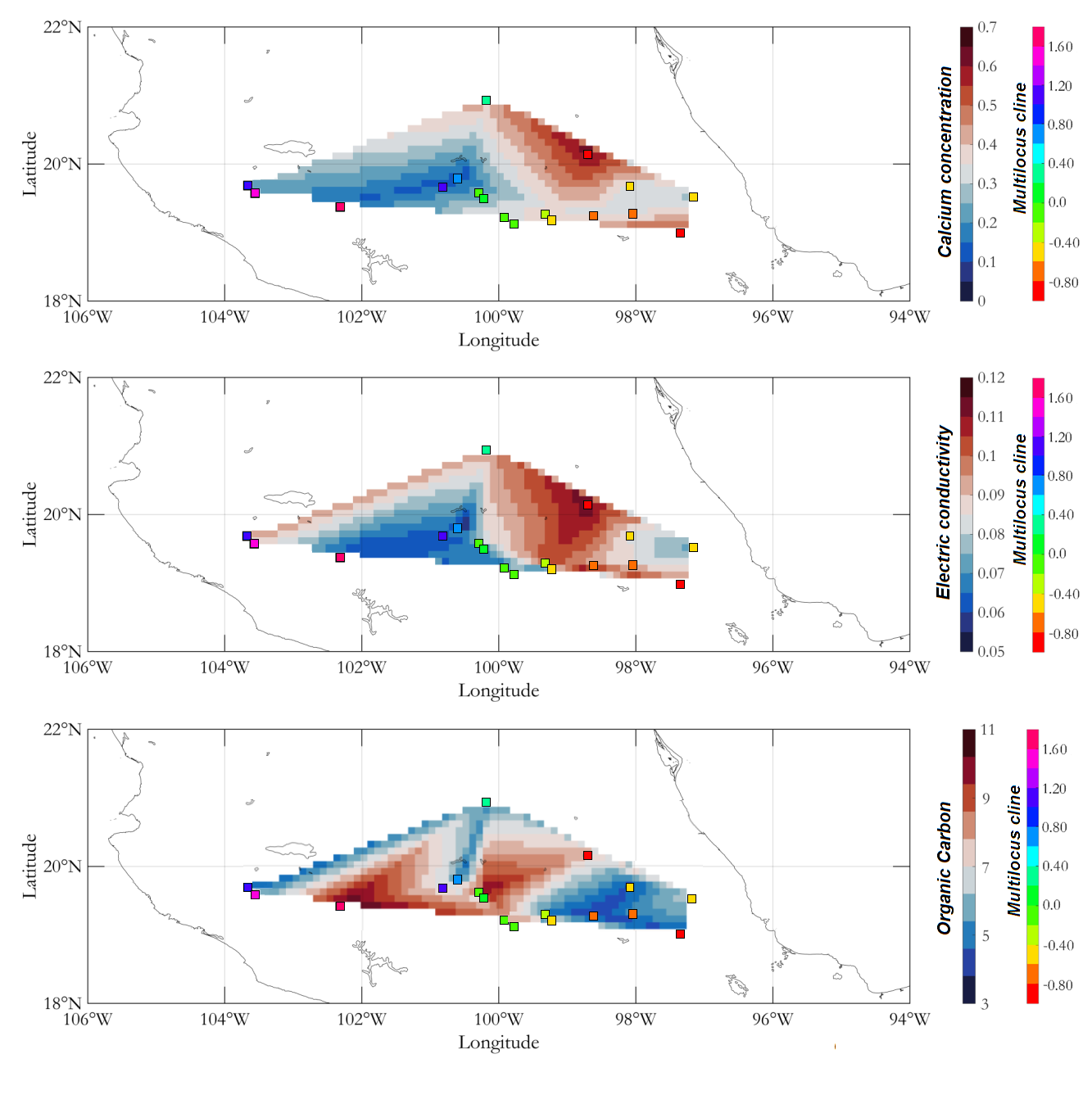


**Suppl. Fig. 6** Spatial distribution of soil calcium concentration, electric conductivity and organic carbon variation in central Mexico and correlation with the population *lagged scores* from a multi-locus cline composed of 49 candidate SNPs in sacred fir (*Abies religiosa*). *Lagged scores* were obtained from a sPCA analysis and reflect the genetic variability linked to the spatial distance between sites.


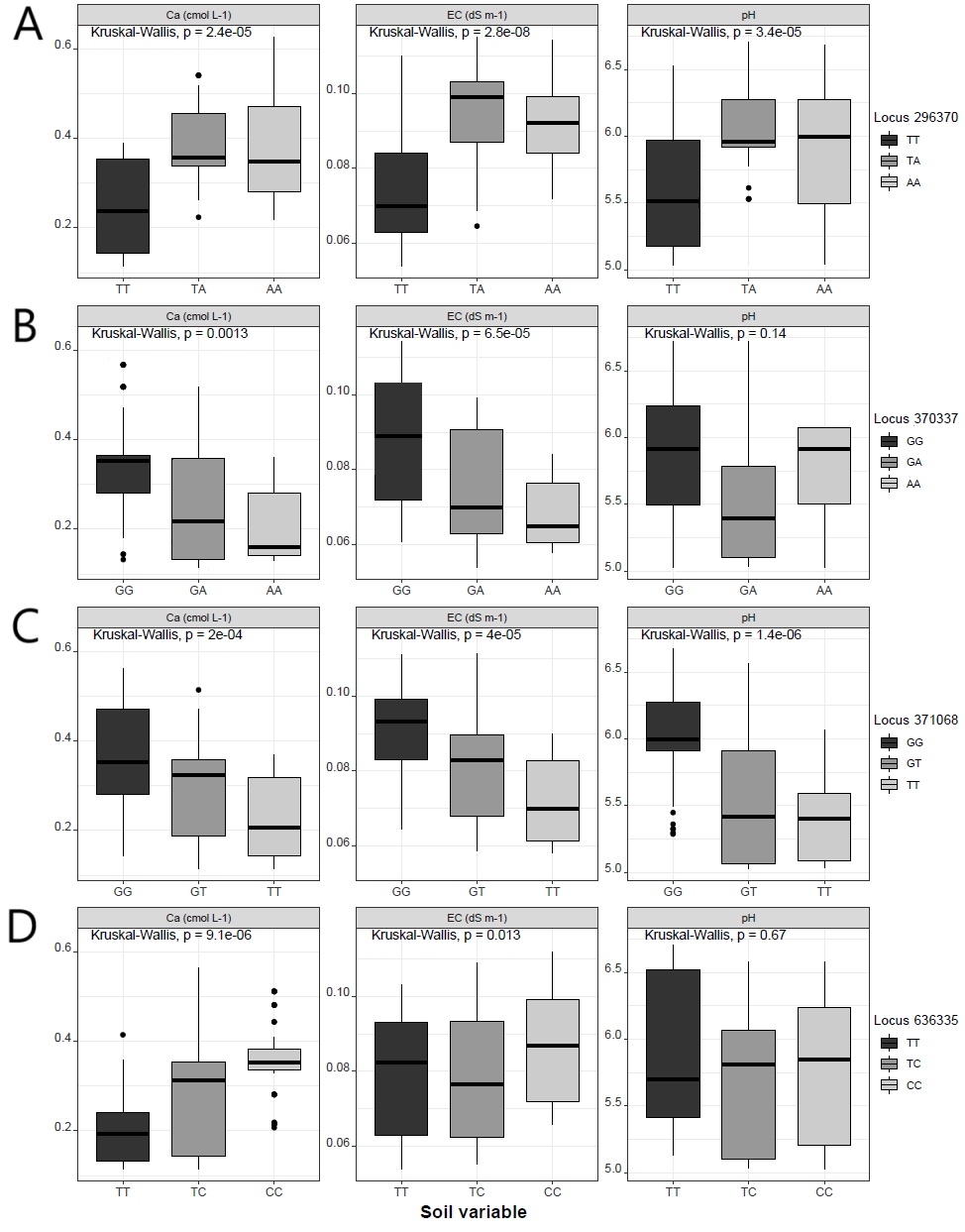


**Suppl. Fig. 7** Significant genotype–environment associations for selected candidate SNPs (*Locus*_296370 (**A**), *Locus*_370337 (**B**), *Locus*_371068 (**C**) and *Locus*_636335 (**D**)) and soil range-wide variation in sacred fir (*Abies religiosa*). Statistical significance according to GEA results (Krustal-Wallis one-way; *P*-value <0.05).).


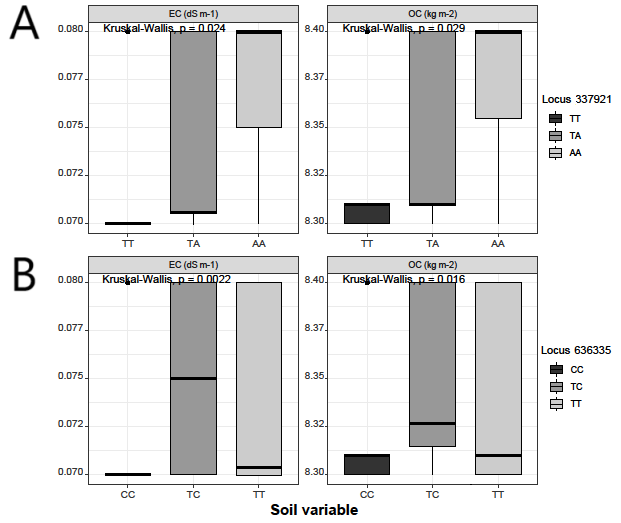


**Suppl. Fig. 8** Significant genotype–environment associations for two candidate loci (*Locus*_337921 (**A**) and *Locus*_636335 (**B**)) and local scale soil variation in sacred fir (*Abies religiosa*). Statistical significance according to GEA results (Krustal-Wallis one-way; *P*-value <0.05).

**SUPPLEMENTARY TABLES**

**Suppl. Table 1.** Sample size, coordinates and estimates of genetic diversity for three *Abies religiosa* stands analyzed at the local scale. Values are also shown for the whole sample. π = Nucleotide diversity, *Ho* = observed heterozygosity and *H_E_* = expected heterozygosity. N corresponds to the number of individuals obtained for each population and N_ES_ to the number of individuals that were efficiently sequenced.

| **Population** | N | N_ES_ | Longitude | Latitude | | Altitude | π | *H_O_* | *H_E_* |
| --- | --- | --- | --- | --- | --- | --- | --- | --- | --- |
| Rincón de Guadalupe (RG) | 29 | 28 | -103.63 | 19.63 | 2515 | | 0.045 | 0.196 | 0.205 |
| High San Bartolo (HSB) | 31 | 26 | -98.08 | 19.68 | 2760 | | 0.046 | 0.199 | 0.205 |
| Down San Bartolo (DSB) | 35 | 33 | -98.04 | 19.26 | 3358 | | 0.049 | 0.208 | 0.210 |
| Whole sample | 95 | 87 |  |  |  | | 0.046 | 0.201 | 0.207 |

**Suppl. Table 2.** Cross-validation results for Admixture analyses of sacred fir (*Abies religiosa*) populations analyzed at the range-wide (A), and local (B) scales.

**A**

| ***K-* value** | ***CV*-error** |
| --- | --- |
| ***K* =1** | 0.4563 |
| ***K* =2** | 0.4348 |
| ***K*=3** | 0.4255 |
| ***K*=4** | 0.4413 |
| ***K*=5** | 0.4558 |
| ***K*=6** | 0.4811 |
| ***K*=7** | 0.4872 |
| ***K*=8** | 0.5073 |
| ***K*=9** | 0.5082 |
| ***K*=10** | 0.5096 |

**B**

| ***K-* value** | ***CV*-error** |
| --- | --- |
| ***K* =1** | 0.509 |
| ***K* =2** | 0.498 |
| ***K*=3** | 0.521 |
| ***K*=4** | 0.550 |
| ***K*=5** | 0.596 |

**Suppl. Table 3A** Pairwise-*F_ST_* matrix for putatively neutral (above diagonal) and candidate SNP variation (below diagonal) among 18 sacred fir (*Abies religiosa*) populations. See Table 1 and Fig. 1 for population names, locations and other details

|  | **SM** | **AJ** | **DL** | **EC** | **NC** | **NT** | **Ix** | **CB** | **PG** | **VT** | **Mo** | **SA** | **Am** | **VA** | **CZ** | **Tl** | **LM** | **CP** |
| --- | --- | --- | --- | --- | --- | --- | --- | --- | --- | --- | --- | --- | --- | --- | --- | --- | --- | --- |
| **SM** | **0** | 0.12 | 0.11 | 0.12 | 0.04 | 0.12 | 0.13 | 0.12 | 0.11 | 0.11 | 0.13 | 0.11 | 0.10 | 0.11 | 0.14 | 0.13 | 0.14 | 0.13 |
| **AJ** | 0.19 | **0** | 0.003 | 0.009 | 0.09 | 0.002 | 0.01 | 0.01 | 0.01 | 0.06 | 0.02 | 0.02 | 0.005 | 0.006 | 0.06 | 0.02 | 0.01 | 0.03 |
| **DL** | 0.16 | 0.03 | 0.00 | 0.02 | 0.09 | 0.01 | 0.02 | 0.01 | 0.02 | 0.06 | 0.03 | 0.03 | 0.01 | 0.02 | 0.06 | 0.02 | 0.02 | 0.04 |
| **EC** | 0.19 | 0.02 | 0.05 | **0** | 0.09 | 0.02 | 0.02 | 0.008 | 0.008 | 0.05 | 0.01 | 0.01 | 0.01 | 0.01 | 0.04 | 0.03 | 0.02 | 0.03 |
| **NC** | 0.06 | 0.15 | 0.14 | 0.19 | **0** | 0.01 | 0.11 | 0.08 | 0.06 | 0.07 | 0.09 | 0.08 | 0.08 | 0.09 | 0.11 | 0.10 | 0.11 | 0.11 |
| **NT** | 0.16 | 0.03 | 0.04 | 0.06 | 0.14 | **0** | 0.01 | 0.006 | 0.01 | 0.06 | 0.01 | 0.02 | 0.001 | 0.01 | 0.05 | 0.02 | 0.02 | 0.04 |
| **Ix** | 0.18 | 0.05 | 0.04 | 0.045 | 0.17 | 0.04 | **0** | 0.02 | 0.03 | 0.08 | 0.03 | 0.03 | 0.02 | 0.01 | 0.06 | 0.02 | 0.015 | 0.04 |
| **CB** | 0.15 | 0.03 | 0.04 | 0.04 | 0.12 | 0.01 | 0.05 | **0** | 0.004 | 0.04 | 0.001 | 0.006 | 0.003 | 0.02 | 0.05 | 0.03 | 0.02 | 0.04 |
| **PG** | 0.14 | 0.16 | 0.12 | 0.20 | 0.09 | 0.11 | 0.17 | 0.11 | **0** | 0.02 | 0.002 | 0.001 | 0.01 | 0.03 | 0.04 | 0.04 | 0.04 | 0.05 |
| **VT** | 0.17 | 0.16 | 0.14 | 0.19 | 0.09 | 0.13 | 0.17 | 0.12 | 0.04 | **0** | 0.04 | 0.04 | 0.05 | 0.07 | 0.06 | 0.08 | 0.08 | 0.09 |
| **Mo** | 0.16 | 0.05 | 0.05 | 0.08 | 0.11 | 0.05 | 0.04 | 0.02 | 0.04 | 0.06 | **0** | 0.001 | 0.01 | 0.03 | 0.04 | 0.04 | 0.04 | 0.05 |
| **SA** | 0.13 | 0.13 | 0.12 | 0.175 | 0.08 | 0.09 | 0.14 | 0.07 | 0.035 | 0.07 | 0.02 | **0** | 0.02 | 0.03 | 0.04 | 0.04 | 0.04 | 0.05 |
| **Am** | 0.16 | 0.03 | 0.05 | 0.08 | 0.12 | 0.02 | 0.05 | 0.01 | 0.11 | 0.13 | 0.03 | 0.08 | **0** | 0.01 | 0.05 | 0.02 | 0.02 | 0.04 |
| **VA** | 0.18 | 0.01 | 0.04 | 0.03 | 0.17 | 0.04 | 0.03 | 0.05 | 0.18 | 0.18 | 0.08 | 0.16 | 0.06 | **0** | 0.05 | 0.01 | 0.01 | 0.01 |
| **CZ** | 0.11 | 0.10 | 0.10 | 0.09 | 0.13 | 0.08 | 0.08 | 0.09 | 0.14 | 0.12 | 0.07 | 0.13 | 0.12 | 0.07 | **0** | 0.06 | 0.06 | 0.05 |
| **Tl** | 0.17 | 0.04 | 0.06 | 0.07 | 0.10 | 0.07 | 0.05 | 0.05 | 0.14 | 0.13 | 0.05 | 0.12 | 0.05 | 0.04 | 0.08 | **0** | 0.03 | 0.04 |
| **LM** | 0.18 | 0.03 | 0.025 | 0.05 | 0.15 | 0.06 | 0.01 | 0.03 | 0.16 | 0.17 | 0.06 | 0.13 | 0.05 | 0.03 | 0.11 | 0.06 | **0** | 0.04 |
| **CP** | 0.20 | 0.05 | 0.06 | 0.07 | 0.15 | 0.08 | 0.07 | 0.06 | 0.17 | 0.15 | 0.08 | 0.14 | 0.06 | 0.03 | 0.11 | 0.05 | 0.06 | **0** |

**Suppl. Table 3B** Significance of the Pairwise-*F_ST_* matrix (*P - values*)

|  | **SM** | **AJ** | **DL** | **EC** | **NC** | **NT** | **Ix** | **CB** | **PG** | **VT** | **Mo** | **SA** | **Am** | **VA** | **CZ** | **Tl** | **LM** | **CP** |
| --- | --- | --- | --- | --- | --- | --- | --- | --- | --- | --- | --- | --- | --- | --- | --- | --- | --- | --- |
| **SM** | NA | 0.000 | 0.000 | 0.000 | 1.000 | 0.000 | 0.000 | 0.000 | 0.000 | 0.000 | 0.000 | 0.000 | 0.000 | 0.000 | 0.000 | 0.000 | 0.000 | 0.000 |
| **AJ** | 0.000 | NA | 0.985 | 0.215 | 0.000 | 0.405 | 0.040 | 0.130 | 0.000 | 0.000 | 0.000 | 0.000 | 1.000 | 0.960 | 0.000 | 0.015 | 0.700 | 0.005 |
| **DL** | 0.000 | 0.987 | NA | 0.495 | 0.005 | 0.850 | 0.500 | 0.490 | 0.000 | 0.000 | 0.000 | 0.000 | 1.000 | 0.970 | 0.000 | 0.250 | 0.810 | 0.000 |
| **EC** | 0.000 | 0.960 | 0.763 | NA | 0.020 | 0.020 | 0.005 | 0.165 | 0.000 | 0.000 | 0.000 | 0.000 | 0.995 | 0.750 | 0.000 | 0.000 | 0.160 | 0.010 |
| **NC** | 0.830 | 0.000 | 0.003 | 0.000 | NA | 0.000 | 0.000 | 0.000 | 0.000 | 0.805 | 0.000 | 0.000 | 0.180 | 0.005 | 0.000 | 0.000 | 0.000 | 0.000 |
| **NT** | 0.003 | 0.620 | 0.480 | 0.107 | 0.000 | NA | 0.045 | 0.180 | 0.000 | 0.000 | 0.000 | 0.000 | 1.000 | 0.605 | 0.000 | 0.010 | 0.425 | 0.000 |
| **Ix** | 0.000 | 0.900 | 0.790 | 0.953 | 0.000 | 0.263 | NA | 0.000 | 0.000 | 0.000 | 0.000 | 0.000 | 0.955 | 0.790 | 0.000 | 0.015 | 0.605 | 0.000 |
| **CB** | 0.000 | 0.757 | 0.753 | 0.257 | 0.020 | 0.907 | 0.340 | NA | 0.000 | 0.000 | 0.000 | 0.005 | 1.000 | 0.230 | 0.000 | 0.000 | 0.115 | 0.000 |
| **PG** | 0.000 | 0.000 | 0.000 | 0.000 | 0.013 | 0.000 | 0.000 | 0.000 | NA | 0.000 | 0.000 | 0.000 | 0.035 | 0.000 | 0.000 | 0.000 | 0.000 | 0.000 |
| **VT** | 0.083 | 0.000 | 0.000 | 0.000 | 0.723 | 0.000 | 0.000 | 0.000 | 0.003 | NA | 0.000 | 0.000 | 0.100 | 0.005 | 0.000 | 0.000 | 0.000 | 0.000 |
| **Mo** | 0.000 | 0.000 | 0.013 | 0.003 | 0.003 | 0.033 | 0.013 | 0.073 | 0.000 | 0.003 | NA | 0.000 | 0.050 | 0.000 | 0.000 | 0.000 | 0.000 | 0.000 |
| **SA** | 0.000 | 0.000 | 0.000 | 0.000 | 0.043 | 0.000 | 0.000 | 0.010 | 0.000 | 0.007 | 0.000 | NA | 0.690 | 0.000 | 0.000 | 0.000 | 0.000 | 0.000 |
| **Am** | 0.000 | 0.807 | 0.563 | 0.087 | 0.027 | 0.890 | 0.467 | 0.990 | 0.000 | 0.000 | 0.147 | 0.023 | NA | 1.000 | 0.000 | 0.940 | 1.000 | 0.060 |
| **VA** | 0.000 | 0.953 | 0.843 | 0.997 | 0.003 | 0.410 | 0.997 | 0.343 | 0.000 | 0.000 | 0.003 | 0.000 | 0.480 | NA | 0.000 | 0.835 | 0.995 | 0.780 |
| **CZ** | 0.000 | 0.000 | 0.000 | 0.000 | 0.000 | 0.000 | 0.007 | 0.003 | 0.000 | 0.000 | 0.000 | 0.000 | 0.000 | 0.007 | NA | 0.000 | 0.000 | 0.000 |
| **Tl** | 0.000 | 0.343 | 0.263 | 0.127 | 0.013 | 0.110 | 0.280 | 0.220 | 0.000 | 0.000 | 0.017 | 0.000 | 0.670 | 0.547 | 0.003 | NA | 0.090 | 0.000 |
| **LM** | 0.000 | 0.840 | 0.920 | 0.773 | 0.010 | 0.490 | 0.997 | 0.647 | 0.000 | 0.000 | 0.013 | 0.000 | 0.927 | 0.983 | 0.000 | 0.527 | NA | 0.000 |
| **CP** | 0.000 | 0.273 | 0.323 | 0.197 | 0.017 | 0.093 | 0.143 | 0.207 | 0.000 | 0.000 | 0.003 | 0.000 | 0.807 | 0.917 | 0.000 | 0.750 | 0.757 | NA |

**Suppl. Table 3C** Pairwise-*F_ST_* matrix for putatively neutral (above diagonal) and candidate SNP variation (below diagonal) among three sacred fir (*Abies religiosa*) populations (stands) at the local scale. See Suppl. Table 1 and Fig. 1 for population names, locations and other details.

| **Population** | RG | HSB | DSB |
| --- | --- | --- | --- |
| RG | 0 | 0.0114 | 0.0089 |
| HSB | 0.0282 | 0 | 0.0043 |
| DSB | 0.0491 | 0.0083 | 0 |

**Suppl. Table 3D** Significance of the Pairwise-*F_ST_* matrix (*P - values*)

| **Population** | RG | HSB | DSB |
| --- | --- | --- | --- |
| RG | 0 | 0.000 | 0.000 |
| HSB | 0.002 | 0 | 0.000 |
| DSB | 0.000 | 0.014 | 0 |

**Suppl. Table 4** Variance partition analysis and significance of polygenic models (edaphic and genomic relationship eigenvalues) for sacred fir (*Abies religiosa*) soil chemical range-wide (**A**) and local variation (**B**) obtained in the db-RDA.

**A**

|  | | ***DF*** | ***Variance*** | ***F*** | ***P-*value** | |
| --- | --- | --- | --- | --- | --- | --- |
| **Model** | | 7 | 3.50 | 3.38 | *** | |
| **Residual** | | 93 | 13.75 |  |  | |
|  | *, *P*-value < 0.05; **, *P*-value < 0.005; ***, *P*-value < 0.001 | | | | |  |

**B**

|  | | ***DF*** | ***Variance*** | ***F*** | ***P-*value** | |
| --- | --- | --- | --- | --- | --- | --- |
| **Model** | | 5 | 1.31 | 1.852 | ** | |
| **Residual** | | 79 | 11.18 |  |  | |
|  | *, *P*-value < 0.05; **, *P*-value < 0.005; ***, *P*-value < 0.001 | | | | |  |

**Suppl. Table 5** Variance partition analysis for a polygenic model and statistical contribution and significance of each individual variable (edaphic) for sacred fir (*Abies religiosa*) soil chemical range-wide (A) and local variation (B) obtained in the db-RDA.

**A**

| **Predictors** | ***Variance*** | ***F*** | ***P-*value** |
| --- | --- | --- | --- |
| ***pH*** | 0.91 | 6.15 | *** |
| ***OC*** | 0.47 | 3.22 | ** |
| ***Ca*^2+^** | 1.03 | 7.02 | *** |
| ***K*^+^** | 0.22 | 1.38 | Ns |
| ***EC*** | 0.53 | 3.41 | *** |
| ***SAR*** | 0.19 | 1.33 | Ns |
| ***Mg*^2+^** | 0.47 | 3.17 | ** |
| **Residual** | 13.75 |  |  |
| *, *P*-value < 0.05; **, *P*-value < 0.005; ***, *P*-value < 0.001 | | | |

**B**

| **Predictors** | ***Variance*** | ***F*** | ***P-*value** | |
| --- | --- | --- | --- | --- |
| ***pH*** | 0.26 | 1.85 | Ns | |
| ***Mg*^2+^** | 0.11 | 0.80 | Ns | |
| ***Ca*^2+^** | 0.32 | 2.26 | * | |
| ***OC*** | 0.29 | 1.97 | * | |
| ***EC*** | 0.37 | 2.58 | ** | |
| **Residual** | 11.19 |  |  | |
| *, *P*-value < 0.05; **, *P*-value < 0.005; ***, *P*-value < 0.001 | | | |  |

**Suppl. Table 6** Variance partition analysis for a polygenic model and statistical contribution and significance of the first five RDA axes accounting for sacred fir (*Abies religiosa*) soil chemical range-wide (**A**) and local variation (**B**) obtained in the db-RDA.

**A**

| **Components** | ***Variance*** | ***F*** | ***P*-value** | |
| --- | --- | --- | --- | --- |
| **dbRDA_1_** | 2.27 | 15.31 | *** | |
| **dbRDA_2_** | 0.56 | 3.73 | ** | |
| **dbRDA_3_** | 0.25 | 1.73 | Ns | |
| **dbRDA_4_** | 0.17 | 1.13 | Ns | |
| **dbRDA_5_** | 0.13 | 0.88 | Ns | |
| **dbRDA_6_** | 0.09 | 0.65 | Ns | |
| **dbRDA_7_** | 0.06 | 0.43 | Ns | |
| **Residual** | 13.75 |  |  | |
| *, *P*-value < 0.05; **, *P*-value < 0.005; ***, *P*-value < 0.001 | | | |  |
|  | | | |  |

**B**

| **Components** | ***Variance*** | ***F*** | ***P*-value** |  |
| --- | --- | --- | --- | --- |
| **dbRDA_1_** | 0.36 | 1.69 | *** |  |
| **dbRDA_2_** | 0.26 | 1.25 | Ns |  |
| **dbRDA_3_** | 0.23 | 1.12 | Ns |  |
| **dbRDA_4_** | 0.19 | 0.93 | Ns |  |
| **dbRDA_5_** | 0.14 | 0.72 | Ns |  |
| **Residual** | 16.08 |  |  |  |
| *, *P*-value < 0.05; **, *P*-value < 0.005; ***, *P*-value < 0.001 | | | | |

**Suppl. Table 7** Results of stepwise multiple regression analyses between the distribution of spatial genetic variation (*lagged scored* 1 and *lagged score* 2) and local soil chemical variation in *A. religiosa*. Abbreviations: EPC, environmental principal component; OC, organic carbon; EC, electric conductivity.

| **Equation Linear Model y = b_0_ + b_1_x_1_** |  | |
| --- | --- | --- |
| **Lagged scores 1** | ***R_adj_^2^*** | ***P – value*** |
| 0.0034 + 0.065 × *Mg*^2+^ | 0.042 | 0.033 |
| 0.0046 + 0.028 × *Ca*^2+^ | - | >0.05 |
| 0.0028 + 0.081 × *pH* | 0.082 | 0.007 |
| 0.0048 + 0.102 × *OC* | 0.082 | 0.004 |
| - 0.0013 + 0.163 × *EC* | 0.155 | 0.0001 |
| 0.0052 + 0.061 × *EPC1* | 0.118 | 0.0006 |
| 0.0047 + 0.004 × *EPC2* | - | >0.05 |
| -0.003 + 0.221 × *EC* – 0.055 × *OC* | 0.152 | 0.0003 |
| **Lagged scores 2** | ***R_adj_^2^*** | ***P - value*** |
| 0.0005 – 0.026 × *Mg*^2+^ | - | >0.05 |
| 0.0004 – 0.015 × *Ca*^2+^ | - | >0.05 |
| 0.0022 – 0.097 × *pH* | 0.056 | 0.015 |
| -0.0009 - 0.068 × *OC* | - | >0.05 |
| -0.0013 + 0.163 × *EC* | 0.134 | 0.0001 |
| -0.0002 - 0.024 × *EPC1* | - | >0.05 |
| -0.0001 - 0.001 × *EPC2* | - | >0.05 |
